# Recent Advances and Prospects in β-type Titanium Alloys for Dental Implants Applications

**DOI:** 10.1021/acsbiomaterials.4c00963

**Published:** 2024-08-31

**Authors:** João V. Calazans Neto, Cícero A. S. Celles, Catia S. A. F. de Andrade, Conrado R. M. Afonso, Bruna E. Nagay, Valentim A. R. Barão

**Affiliations:** 1 Department of Prosthodontics and Periodontology, Piracicaba Dental School, Universidade Estadual de Campinas (UNICAMP), Piracicaba, São Paulo 13414-903, Brazil; 2 Department of Materials Engineering (DEMa), Universidade Federal de São Carlos (UFSCar), São Carlos, São Paulo 13565-905, Brazil

**Keywords:** Titanium alloys, Implants, Mechanical properties, Elastic modulus, Corrosion resistance

## Abstract

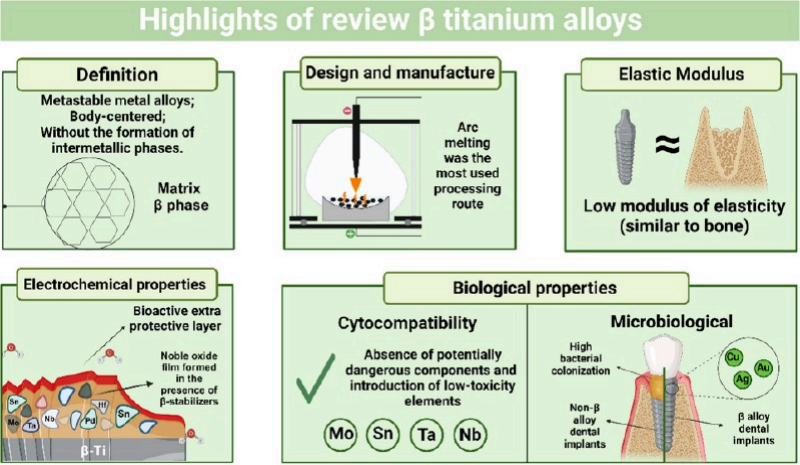

Titanium and its alloys, especially Ti-6Al-4V, are widely studied in implantology for their favorable characteristics. However, challenges remain, such as the high modulus of elasticity and concerns about cytotoxicity. To resolve these issues, research focuses on β-type titanium alloys that incorporate elements such as Mo, Nb, Sn, and Ta to improve corrosion resistance and obtain a lower modulus of elasticity compatible with bone. This review comprehensively examines current β titanium alloys, evaluating their mechanical properties, in particular the modulus of elasticity, and corrosion resistance. To this end, a systematic literature search was carried out, where 81 articles were found to evaluate these outcomes. In addition, this review also covers the formation of the alloy, processing methods such as arc melting, and its physical, mechanical, electrochemical, tribological, and biological characteristics. Because β-Ti alloys have a modulus of elasticity closer to that of human bone compared to other metal alloys, they help reduce stress shielding. This is important because the alloy allows for a more even distribution of forces by having a modulus of elasticity more similar to that of bone. In addition, these alloys show good corrosion resistance due to the formation of a noble titanium oxide layer, facilitated by the incorporation of β stabilizers. These alloys also show significant improvements in mechanical strength and hardness. Finally, they also have lower cytotoxicity and bacterial adhesion, depending on the β stabilizer used. However, there are persistent challenges that require detailed research in critical areas, such as optimizing the composition of the alloy to achieve optimal properties in different clinical applications. In addition, it is crucial to study the long-term effects of implants on the human body and to advance the development of cutting-edge manufacturing techniques to guarantee the quality and biocompatibility of implants.

## 1 Introduction

The quest for materials suitable for biomedical applications, particularly in dental implants, remains a challenge in Implantology, driven by an aging population and the high incidence of failure rates, thereby increasing the demand for successful rehabilitation treatments.^1−3^ In this pursuit, the ideal material must exhibit a balance of durability and exceptional mechanical properties while demonstrating robust corrosion resistance and biocompatibility.^1^ Achieving this balance necessitates a comprehensive understanding of the material’s intrinsic properties, which can be predicted through meticulous modeling and tailored processing techniques, including postprocessing methods that influence microstructural outcomes.^2,4^ Therefore, a deeper insight into the material’s composition and structure is crucial for optimizing its performance in biomedical applications. Moreover, due to their exposure to the oral environment and physiological fluids, dental implants are susceptible to corrosive processes, undergoing passive degradation and deterioration, necessitating specialized corrosion mitigation mechanisms and techniques.^2,5^ Once the inherent electrochemical oxidation of titanium facilitates the formation of a protective layer of passive titanium dioxide (TiO_2_) on the material’s surface,^6^ the microstructure, surface quality, and composition of a material play crucial roles in determining its corrosion resistance, highlighting the importance of these factors in dental implant materials.

**Figure 1:**
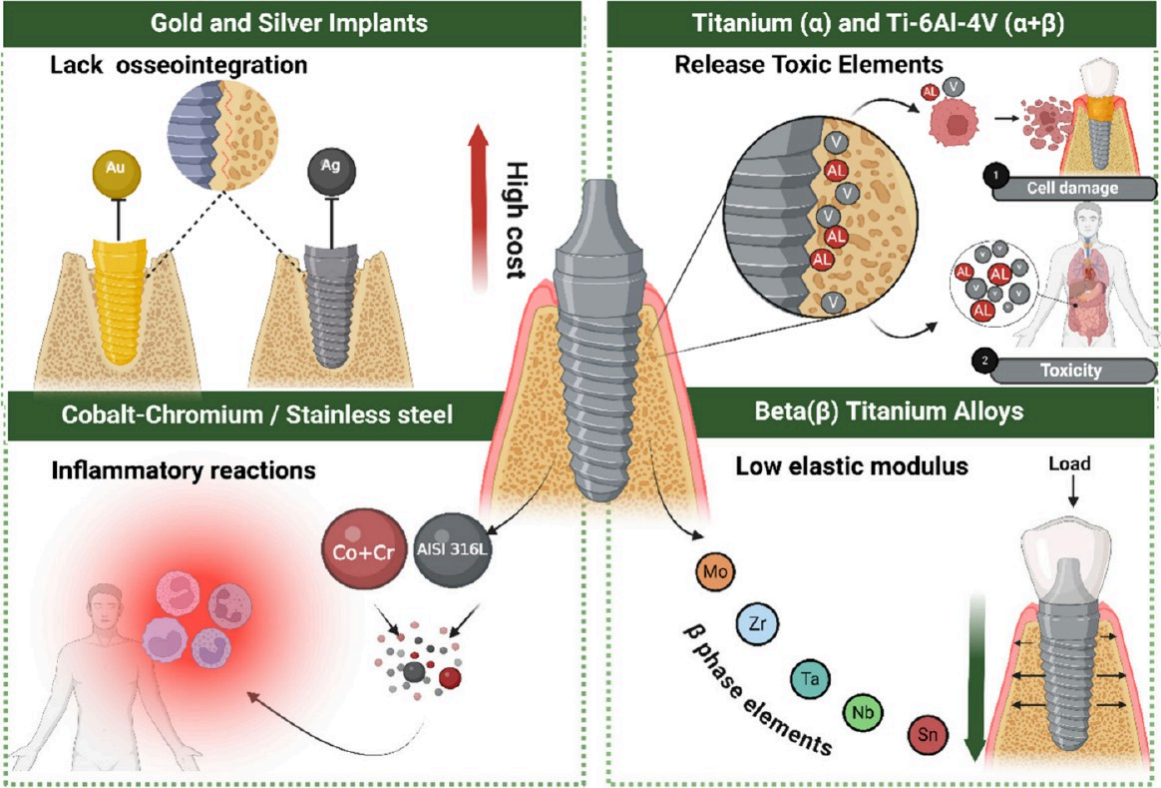
Materials in dental implants and their drawbacks: Gold and silver lack osseointegration and are expensive. Cobalt-chromium and stainless steel (AISI 316L) cause inflammatory reactions. Titanium (α) and Ti-6Al-4V release toxic elements like aluminum (Al) and vanadium (V), damage cells, and have systemic toxicity. B titanium (β) alloys (Mo, Zr, Nb, Ta, Sn) have lower modulus, better load distribution, and potentially better osseointegration. The figure was created with BioRender.com (License number: ZU272R4OA5).

Different materials have been used in the production of implants, each with its advantages and disadvantages (Figure 1). In the past, precious metals such as gold, silver, and platinum were widely used. However, due to their high cost and limitations such as inadequate mechanical properties, variable biological compatibility, and difficulties in manufacturing, these materials have been replaced by more affordable metallic options with more suitable characteristics for the application.^7^ Even in the past, in the search for alternatives that offer similar performance, the industry turned to base metals such as cobalt-chromium (Co-Cr) alloys and stainless steel. Co-Cr alloys are known for their high mechanical strength, and resistance to wear and corrosion.^8,9^ However, the cobalt content can release harmful ions and cause inflammatory reactions in some patients.^10^ Stainless steel, especially the AISI 316L type, was cost-effective compared to other alloys.^11^ However, under specific aggressive physiological conditions, localized corrosion and erosion can occur, resulting in the release of toxic metal ions.^12,13^

Titanium (Ti) and its alloys are extensively researched and utilized in biomaterial applications due to their unique combination of physical, chemical, and mechanical properties. These include corrosion resistance, high strength-to-weight ratio, fatigue and traction resistance, and biocompatibility with peri-implant tissues,^14−16^ which are intricately linked to the micro and macrostructure of the material, dictating its behavior and potential for optimization in large-scale applications.^17−19^ Specifically, Ti alloys, categorized into alpha (α), β (β), and α+β structures based on their polymorphic phases, exhibit diverse properties influenced by their structure and exposure temperature.^20^ Among these alloys, the Ti-6Al-4V (α+β) alloy stands out as predominant in the dental implant market. However, it faces limitations such as concerns about cytotoxicity, i.e., its potential to cause damage or cell death due to the release of toxic elements into biological tissues. Additionally, its elastic modulus (∼110 GPa) is considered high compared to human cortical bone (10–40 GPa), which can lead to stress shielding and subsequent bone atrophy due to the mismatch between these values.^1,2,21−24^

In this context, to address those above mechanical and electrochemical limitations, extensive research has focused on optimizing the Ti materials through the formulation of new alloys, incorporating β phase elements like molybdenum (Mo), niobium (Nb), tin (Sn), zirconium (Zr) and tantalum (Ta), along with thermo-mechanical treatments. For example, the controlled addition of Zr and Nb enhances wear resistance and shows potential for strengthening the microstructure of alloys.^22,25−31^ By altering these materials’ composition, processing, and deformation, it becomes possible to transition from a compact hexagonal to a cubic body-centered crystalline structure.^32^ This structural transformation increases the distance between atoms, facilitating movement and creating more empty spaces susceptible to elastic and shape memory effects due to lower structural rigidity and elastic modulus.^5,22,33,34^ In terms of corrosion resistance, the addition of β-stabilizers helps the prevention of the occurrence of the microgalvanic effect by balancing the electrochemical potential of materials and promoting the formation of stable oxides. Consequently, this enhances phase stability, structural integrity, and the development of thicker and nobler passive layers acting as protective barriers.^15,35^ Thus, by combining β elements within alloys, their respective advantages are leveraged, yielding less allergenic and toxic materials while exhibiting enhanced biocompatibility and suitability for biomedical applications.^22,27−31^

Their composition and manufacturing methods influence the properties of β-type titanium (Ti) alloys. Additive manufacturing (AM) methods, such as Selective Laser Melting (SLM), combined with postprocessing heat treatments and advanced techniques like high-energy milling and plasma sintering, have revolutionized the production of β-titanium (β-Ti) alloys by enabling the fine-tuning of their microstructure and properties, resulting in enhanced yield strength, fatigue resistance, and wear resistance.^36−38^ This technological progress has led to extensive research and widespread utilization of β-type Ti alloys, with a focus on achieving desired characteristics such as high strength, and enhanced ductility. This is done through careful control of interstitial oxygen levels, premartensitic or anomalous softening, and stabilization of β elements while suppressing undesirable phases like ω, which can degrade the mechanical properties of the alloy, leading to structural instability, compromising machinability and, in some cases, negatively impacting corrosion resistance.^23,39^ Nevertheless, the fabrication of biomaterials using β-type Ti alloys remains challenging due to various factors, such as the high cost associated with large-scale production, the scarcity of β-stabilizing elements for future applications, and the intricate mechanisms involved in materials science and metallurgy, which necessitate a comprehensive understanding of effective parameters.^40^ Also, difficulties arise in the casting process, and a mechanical mismatch often occurs between the biomaterial and the surrounding bone tissue.^20,41,42^ Thus, to address these issues, researchers have explored the incorporation of lower-cost elements such as Iron (Fe), Manganese (Mn), and Chromium (Cr), which also serve to stabilize the β-phase.^20^ Furthermore, strict control over processing conditions, precise heat treatment protocols, and careful material selection are imperative to achieve the desired and efficient properties in β-type Ti alloy biomaterials.

Previous literature reviews^1,43−45^ have highlighted the promising attributes of β-type Ti alloys, underscoring the ongoing research, innovation, and utilization of these materials in biomedical applications to enhance performance. However, these reviews often concentrate on specific alloys and manufacturing methods,^46^ along with orthopedic implants,^47,48^ while overlooking applications specific to dental implants. This oversight can lead to gaps in understanding the current state of the art and create opportunities for future research in this specialized area. Therefore, our comprehensive review aims to critically evaluate the impact of β-type Ti alloys on the mechanical and biological properties of implants. To offer a structured analysis, the review commences with a narrative exploration of the structure of β-type Ti alloys, manufacturing methods, and the factors influencing their synthesis, mechanical, and biological properties. Subsequently, a systematic summary of existing studies described variations in alloy composition, associated elements, and other conditions impacting their properties, including elastic modulus and corrosion resistance. Finally, the review offers insights into current literature trends and emerging challenges in the field.

## 2 Materials Used to Manufacture Dental Implants

Over the past few decades, the materials utilized for dental implant manufacturing have undergone significant evolution.^49^ In antiquity, natural materials like animal bones were often used to replace lost teeth.^50^ During the Middle Ages, advancements in metallurgy led to the utilization of metals such as gold and silver.^50^ However, these materials had limitations, including a lack of osseointegration and biocompatibility, resulting in implant failure.^51^ The true revolution in dental implant history occurred in the 20th century with the discovery of Ti potential.^52^ From that time, the evolution of dental implant materials has been driven by technological advancements and the quest for materials that meet the key requirements necessary for successful integration into bone tissue.

The criteria for an ideal bone implant material include biocompatibility, corrosion resistance, strength, wear resistance, and elastic modulus.^53^ Thus, selecting the appropriate material for dental implants is critical as it must withstand masticatory forces, provide long-term stability, and exhibit biocompatibility to prevent rejection by the host.^54^ An essential consideration is the similarity of the material’s mechanical properties to natural bone, such as the elastic modulus, to ensure proper load distribution during masticatory function.^4^ Considering that the design of the implant should be compatible with the inherent physical characteristics of the chosen material, dental implant materials can be categorized according to their chemical composition, including metals, ceramics, and polymers, each possessing distinct characteristics and properties.^55,56^

In addition to metallic materials, ceramics like zirconia and alumina also offer distinct advantages in dental implants. Zirconia, with its high biocompatibility and resistance to wear, is aesthetically pleasing and resistant to corrosion implants.^55,57^ Commercially available systems such as Straumann Ceramic provide dental implant solutions featuring zirconia components. On the other hand, Alumina exhibits excellent tribological behavior but has limitations in fracture toughness and stress deformation.^57^ Polyether ether ketone (PEEK) is another material that is gaining interest in dental implant applications due to its mechanical properties, which are similar to bone.^58^ However, challenges with osseointegration exist, necessitating surface treatment techniques for improvement.^58^

Despite the advancements in materials like ceramics and PEEK, Ti alloys remain a cornerstone in dental implant manufacturing due to their exceptional cost-benefit properties. Among the most widely used Ti alloys are Ti-6Al-4V and Ti-15Zr.^59^ Nevertheless, their high elastic modulus and potential cytotoxicity have driven the search for alternative Ti alloys. β-type Ti alloys have emerged as promising solutions, addressing these challenges and advancing dental implant technology.^60,61^ These properties reduce the risk of stress shielding and adverse tissue reactions, ultimately promoting better long-term implant stability and patient comfort. As a result, β-type Ti alloys have been increasingly recognized as essential components in modern dental implant solutions, aiming to provide clinicians with more reliable options for successful implantation and improved patient care soon.

## 3 β-Type Titanium Alloys

### 3.1 Definition and Characteristics

**Figure 2:**
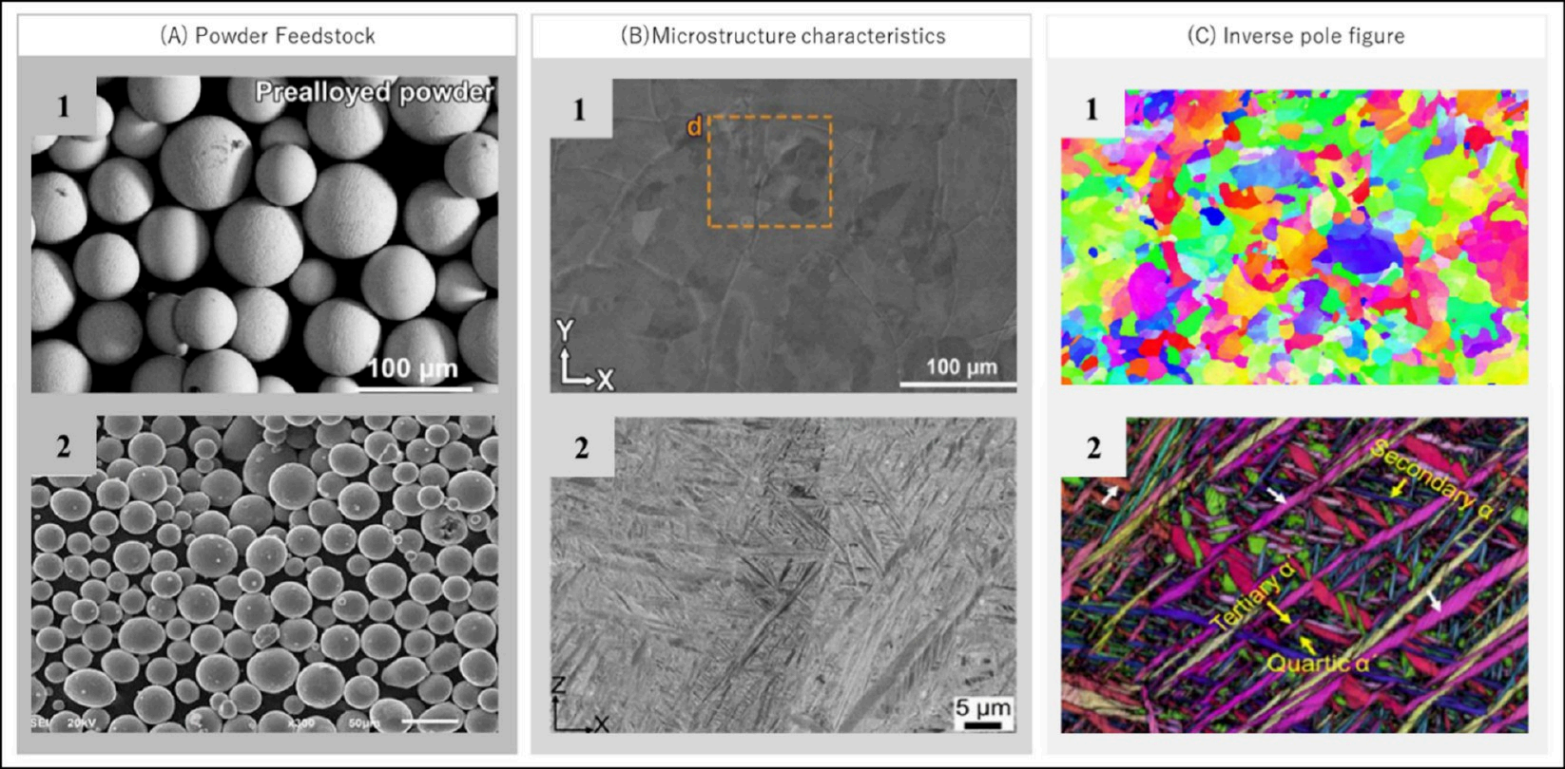
(A1) shows the shape of the prealloyed Ti-35Nb powder after the mixing process. (A2) shows the prealloyed Ti-6Al-4V alloy powder, which was also manufactured by SLM. (B1) shows the microstructure of Ti-35Nb, which contains equiaxed β grains and no α precipitates. (B2) illustrates the microstructure of Ti-6Al-4V, highlighting the presence of acicular α′ martensite, as a result of rapid cooling during the SLM process. (C1) shows an EBSD analysis which reveals the presence of columnar and equiaxed β grains formed in different directions of construction. (C2) shows the secondary α′ martensite in the Ti-6Al-4V alloy, aligned with or perpendicular to the primary α′ martensite, as well as the presence of tertiary and quartic phases. Reproduced or adapted with permission from [^38^]. Copyright [2022] [Elsevier]. Reproduced or adapted with permission from [^64^]. Copyright [2023] [MPDI].

β-type Ti alloys are a subset of Ti alloys characterized by their distinctive microstructural properties and composition. Unlike α-β Ti alloys, which contain both α and β phases, β-type Ti alloys consist predominantly or entirely of the β phase. This β phase exhibits a body-centered cubic (bcc) crystalline structure and remains stable at temperatures above 880 °C.^61,62^ The microstructure of β-type Ti alloys is characterized by asymmetrical grains and acicular structures in intragranular regions. The complete constitution of a pre-alloyed β alloy, Ti-35Nb, compared to a biphasic α + β alloy, Ti-6Al-4V, is shown in Figure 2. It highlights the microstructural differences between them and shows how variations in compositions and manufacturing processes influence these differences, particularly in the distinct characteristics of grains and crystallographic phases present in each material. The combination of different elements generates a characteristic heterogeneity in the prealloyed β alloy material, which has spherical powders and a very smooth surface, as reported by the authors^38^ and illustrated in Figure 2 A1. This contrasts with the prealloyed Ti-6Al-4V powders, shown in Figure 2 A2. Although the processing method for both is similar, employing the Selective Laser Melting (SLM) technique, the differences in crystallographic compositions lead to marked variations in the microstructure (Figures 2 B1 and 2 B2), in which the prealloyed β alloy powder has a highly homogeneous microstructure with β grains and evident grain contours (Figure 2 B1). In addition, mapping of the crystallographic phases by electron backscattered diffraction (EBSD) reveals the formation of columnar and equiaxed β grains, arranged in the directions parallel and perpendicular to the build, for the β alloy (Figure 2 C1). These β grains are highly homogeneous in the horizontal scan plane, indicating random orientations.38 In contrast, the Ti-6Al-4V alloy exhibits a variety of α′ Martensite phases, as shown in Figure 2 C2.

Regarding the composition, β-type Ti alloys are usually enriched with β-stabilizing elements such as Mo and Ta while containing fewer α-stabilizers, thereby avoiding the formation of intermetallic phases.^61,62,65,66^ Additionally, β-type Ti alloys are primarily comprised of transition metals, categorized as isomorphous or eutectoid based on their influence on the titanium matrix, and may contain secondary platelets of the α hexagonal close-packed (hcp) phase or particles formed during isothermal aging.^60^

One of the main advantages of β-type Ti alloys is their unique mechanical properties and corrosion resistance, making them highly desirable for various applications, including dental implants, aerospace components, and biomedical devices. Due to the absence of microgalvanic interactions among different phases, β-type Ti alloys are anticipated to offer superior corrosion resistance within the human body compared to α+β alloys.^40,67^ Moreover, they provide comparable strength and enhanced biocompatibility, making them an excellent choice for implants where stability, durability, and compatibility with the surrounding tissue are paramount.^40,67^ To achieve such properties, these alloys can undergo heat treatment processes involving solution conditioning and aging at temperatures ranging from 450° to 650 °C, which enhances their strength through dispersion strengthening arising from the partial transformation of the β to the α phase.^48,68,69^ By increasing the proportion of the β phase, these alloys demonstrate improved toughness, plasticity, and age hardening, accompanied by a reduction in elastic modulus.^48,68,69^

With a lower Young’s modulus, β-type Ti alloys closely match the modulus of human bone, minimizing bone degradation and absorption. This characteristic may enhance the longevity and success of dental implants.^70−72^ Furthermore, β-type Ti alloys show promise in enhancing bone regeneration and remodeling, aiding in the effective integration of prostheses and lowering the risk of implant failure.^70,71,73^ They also offer superior biocompatibility by preventing the release of metal ions, which is associated with long-term health issues.^70,74,75^ Furthermore, these alloys exhibit excellent in vivo osseointegration characteristics, minimizing the risk of implant-induced oxidative stress and subsequent inflammatory activation that may occur with other alloy compositions.^67,76^

Given their numerous advantages and ability to provide precise control over processing variables, β-type Ti alloys have sparked significant interest in structural applications. Despite their relatively high costs, they are increasingly considered viable alternatives for replacing rigid tissues, particularly in medical prostheses and dental implants. This is due to their exceptional properties, such as low elastic modulus, which closely mimic the properties of bone, making them well suited to such applications.^69,70,73,77^ Nevertheless, despite their promising potential, these alloys face limitations, including low fatigue strength and hardness due to the micrometric size of their grains, which underscores the necessity for further exploration.^78^

### 3.2 Composition Matters: Alloying Materials for Implant Manufacturing

As previously mentioned, in the realm of Ti alloy composition, adjustments in the type and quantity of elements play a pivotal role, leading to the classification of these alloys into four main groups: α, near-α, (α + β), and β alloys.^48,79^ β-phase alloys are characterized by stabilizing elements such as Mo, referred to as β-stabilizers, which are transition elements.^48,67,80^ The design of materials within this alloy category primarily revolves around the stability of the β phase, assessed through parameters like molybdenum equivalence (Moeq). For example, elements like Nb and Cr contribute to reducing the Moeq value, indicating their positive influence on the stability of the β phase in Ti alloys.^48,69,81^ These elements also exert significant influence on the microstructure of the material, consequently impacting the wear mechanism of these innovative alloys. For instance, the incorporation of Nb in Ti-xNb-7Fe alloys, with varying Nb content ranging from 0.5% to 9% by weight, increases the β phase proportion in the microstructure, resulting in decreased wear resistance.^82^

Besides structural properties, the pursuit of cost-effective yet mechanically advantageous materials has fueled innovation in recent years. Initially, alloys like Ti-12Mo-6Zr-2Fe (TMZF), Ti-15Mo, Ti-15Mo-5Zr-3Al, Ti-35.3Nb-5.1Ta-7.1Zr (TNZT), and Ti-29Nb-13Ta-4.6Zr (TNTZ) were developed, albeit facing challenges due to the scarcity of elements and high production costs.^74,83^ They also have high melting points, contributing to segregation and presenting challenges in the material preparation process.^74^ Since then, β-type Ti alloys have evolved to include cost-effective elements like Fe, Cr, Mn, and Sn, aiming to reduce costs and eliminate the use of toxic elements while maintaining low modulus and advantageous mechanical properties for biomedical applications.^48,74,81,84^ These alloys, exemplified by compositions such as Ti-10Cr-Al, Ti-Mn-Fe, Ti-Mn-Al, Ti-Cr-Al, Ti-Sn-Cr, Ti-Cr-Sn-Zr, and Ti-(Cr, Mn)-Sn, are exclusively comprised of alloying elements naturally found in the human body or with no identified adverse health effects.^74,85,86^ This shift toward more accessible alloy compositions addresses cost concerns and underscores a commitment to enhancing dental implants’ biocompatibility and safety profile and other biomedical devices.

Typically, producing these β-type Ti alloys involves creating a polycrystalline structure with a random texture, which helps maintain a single β-phase microstructure during rapid cooling from high temperatures.^48^ This manufacturing method favors isotropic properties, which are essential for biomedical applications, where uniformity of mechanical properties is key. The search for a lower elastic modulus in such alloys aims not only to improve biocompatibility, allowing better adaptation to the biological environment but also to provide a higher strength-to-weight ratio, which is advantageous for implants that must withstand mechanical loads.^48,71,87^ In addition, corrosion resistance is fundamental to the durability of these materials in the human body, while resistance to strain-controlled and notch-controlled fatigue is crucial to ensuring structural integrity over time.^67,84^ Overall, the search for specific properties in these alloys, combined with the challenges of manufacturing and biomedical requirements, highlights the importance of carefully balancing the desired characteristics. The optimization of these alloys aims not only to meet biomechanical requirements but also to consider economic aspects, minimizing adverse effects to promote their accessibility and safety in future biomedical applications.^71,81,88^

### 3.3 From Production to Precision: Manufacturing Methods and Post-Processing Techniques

#### 3.3.1 Manufacturing Methods

**Figure 3:**
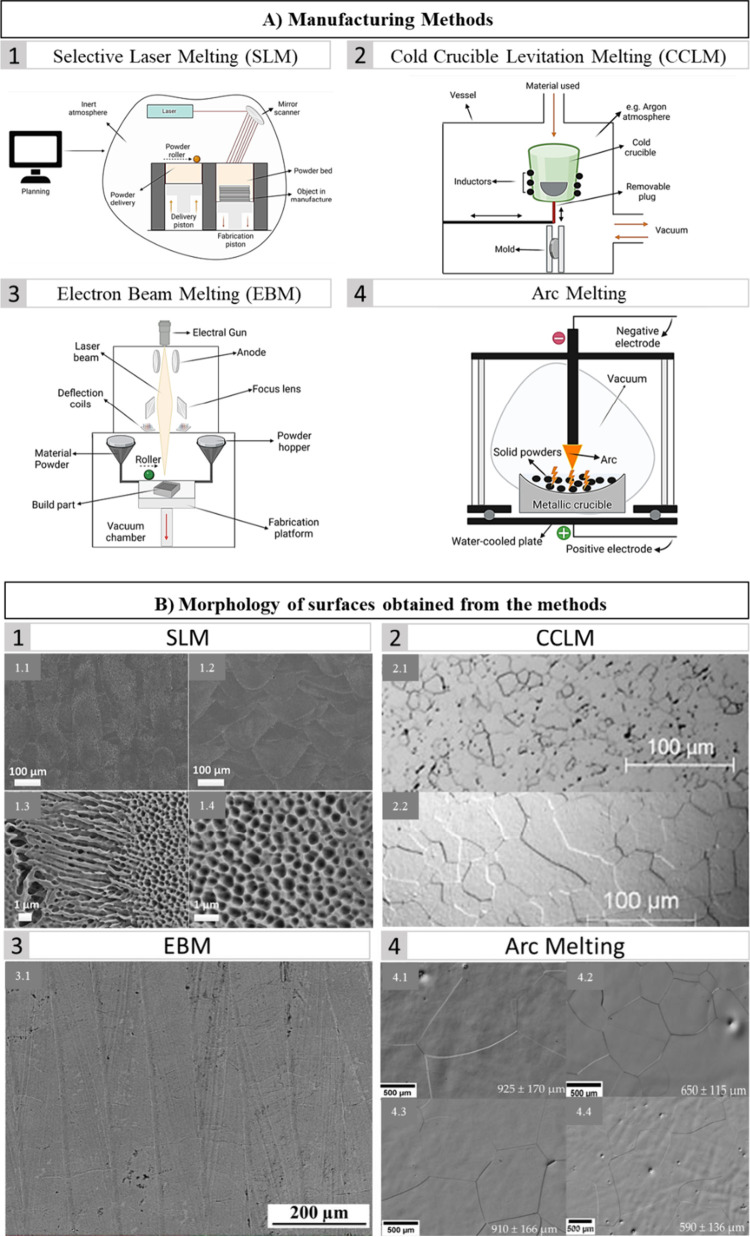
Schematic illustration depicting production methods for creating biomaterials from various β-titanium alloys (top panel), followed by representative micrographs showcasing surfaces obtained through these methods (bottom panel). (A) Depiction of processing methods, including (A1) selective laser melting, (A2) cold crucible levitation melting, (A3) electron beam melting, and (A4) arc melting. (B) Micrographs displaying the morphology of surfaces obtained from the methods of SLM (B1)93; CCLM (B2)91; EBM (B3)75; and arc melting (B4)89. Image A was created with BioRender.com (License number: WU272DD911, UK272DD9AN, PM272DD9DL, and BV272DD9GZ). Reproduced or adapted with permission from [^91, 93^]. Copyright [2010, 2019] [Elsevier]. Reproduced or adapted with permission from [^89^]. Copyright [2023] [MPDI].

With the increasing demand for advanced biomedical materials, particularly in dental and orthopedic implants, the manufacturing methods and postprocessing techniques of β-type Ti alloys have garnered significant attention. Despite the advantageous mechanical properties, enhanced corrosion resistance, and biocompatibility exhibited by β-type Ti alloys, one of the challenges that still need to be addressed is their high processing costs. The choice of processing method directly affects the properties of the materials, influencing the variety of possible microstructures. β-type alloys offer flexibility in chemical adjustments, combinations of elements, and processing options, resulting in promising mechanical and biocorrosion properties. These materials can be produced by different methods, such as casting, melting, and sintering. However, casting has disadvantages, such as material waste and limitations in geometry. At the same time, additive manufacturing stands out for allowing customization, conservation of raw materials, and the creation of complex shapes, which can improve the precision of medical implants and reduce costs.^75,89−91^ For the production of biomaterials with β titanium alloys, the most commonly reported methods are arc melting, powder metallurgy, hot forging, cold crucible levitation melting (CCLM), selective laser melting (SLM), electron beam melting (EBM), and spark plasma sintering, as illustrated in Figure 3.^75,89−91^ These techniques offer versatility in tailoring the microstructural characteristics of β-type Ti alloys, thereby influencing their mechanical properties and performance in biomedical applications (Figure 3B). Following processing, these alloys usually undergo postprocessing procedures, such as heat treatment, to tailor their microstructure and strength to desired levels.^48,60^ This occurs mainly because thermomechanical treatment can enhance mechanical properties, mitigating residual stresses, increasing ductility, preventing the formation of undesirable phases such as ω and α around β grain boundaries, and improving corrosion resistance.^60,92^

Among processing methods, additive manufacturing has emerged as a revolutionary technique, offering unparalleled flexibility in geometric complexity and composition control and simplifying processes to maximize production and increase the applicability of materials in different areas.^94^ This innovative method enables the fabrication of intricate structures through a layer-by-layer process, allowing for precise adjustments in material composition and parameters, with customization of the final object given the energy source used in the process to melt the metal powder.^95,96^ In this technique, as layers are sequentially added and fused, the platform descends, paving the way for the formation of subsequent layers until the final object is synthesized.

For a successful additive manufacturing process, establishing the right parameters is crucial to producing parts that perform mechanically and structurally without compromising their application. Thus, parts with heteromorphic surfaces and coarse finishes, the presence of unmelted or partially melted powder particles, and also disunity between layers are the main defects associated with the unstable formation of the melt pool, the area of molten material created by the laser.^64,94,97^ This instability of the molten pool, generated by the high temperatures and abrupt cooling inherent in the process, has a direct impact on the mechanical properties and corrosion resistance of the materials. In this context, knowledge of the need to adjust ideal parameters, such as laser power, layer thicknesses, scanning speed, and metal powder properties, will dictate the wetting condition of this pool, which may present undesirable results of defects due to holes or bubble formation with impacts on the microstructure and properties of the material.^64^ In this way, we want parameters that promote a strong bond between the layers and a stable melting pool that favors this connection and prevents bubbles, spatters, and melting heterogeneity.

An advance that predicts manufacturing success is the possibility of simulations with melt pool models capable of visualizing the microstructure and the possible formation of defects, reducing or zeroing out the chances of parts with incomplete melting, undesirable pores, residual stress, cracks, and compromised mechanical properties.^64^ A complete simulation involves considering the aforementioned parameters and allows for the development of robust and reliable models, with greater precision and standardization.^64,98^ Among the various additive manufacturing techniques, SLM and EBM stand out as the most prominent methods for producing β-type Ti alloys. SLM employs a high-power laser in an inert atmosphere, facilitating rapid production of consecutive layers based on computer-aided design (CAD) specifications, with high surface quality and precision.^99^ Conversely, EBM operates within a vacuum chamber, utilizing an electron beam to selectively melt metal powder, and although is typically limited to producing metallic components, it remains a valuable technique for fabricating β-type Ti alloy structures.^99^ When comparing SLM with EBM in terms of susceptibility to defects, it can be seen that SLM has a lower platform temperature, which causes the surface tension of the molten pool to increase rapidly as the temperature decreases, resulting in the formation of pores. In contrast, in EBM, the platform operates at higher temperatures and has slower cooling rates due to the vacuum environment. This allows the consolidated layers to maintain a constant temperature, protecting the process from the formation of deep holes and other defects.^64^

However, it is crucial to note that the thermal cycles inherent in additive manufacturing can induce phase changes in materials, affecting their mechanical properties. For low-modulus β-type Ti alloys, rapid cooling is essential to preserve the desired β phase.^92,100^ Nevertheless, it is important to note that challenges remain in the additive manufacturing of these alloys, particularly concerning elements with high melting points, known as β-refractory stabilizers. Elements such as Nb, Ta, and Mo may not melt completely during processing, leading to their partial dispersion in the alloy and hindering homogeneity.^22,101,102^ In evaluating the potential of Mo, an element from the β-isomorphous family, Zhang et al., 2024^103^ when forming Ti-5Al-5Mo-5V-3Cr-5Mo alloys observed that Mo acted as nuclei enabling crystal formation and refinement during the formation of the layers, as well as stabilizing the desired β phase without phase heterogeneity in thermal cycles. Consequently, resolving the limitations is fundamental to guaranteeing the integrity and uniformity of the final product, thus maximizing the potential of additive manufacturing for the production of β-type Ti alloys.

Ummethala et al., 2020^95^ reported positive results using SLM, in which the production of devices in the Ti-35Nb-7Zr-5Ta alloy showed an elastic modulus of approximately 81 GPa as well as excellent mechanical strength, which were attributed to the rapid cooling inherent in the SLM process, promoting the formation of a cubic body-centered crystalline structure with fine, narrow, and elongated grains. Corroborating these results in terms of the versatility of SLM, Fisher et al., 2016^102^ conducted an in situ alloy study in which they reported the versatility of SLM in adjusting elemental compositions, resulting in homogeneous and compact alloys. Regarding EBM, Kozadaeva et al., 2023^75^ presented pioneering results utilizing such a method, reporting a low elastic modulus for the Ti-42Nb alloy along with promising yield strength and excellent anticorrosion properties. These findings further underscore the potential of additive manufacturing techniques in the fabrication of advanced materials.

While additive manufacturing techniques offer notable advantages in the fabrication of β-type Ti alloys, such as precise control over geometric complexity and composition, they still face limitations, such as cost-effectiveness and homogeneity. As an alternative, devices can also be produced using arc melting, a technique conducted in a high-temperature furnace. This method involves heating metals with an electric arc formed by metal electrodes, resulting in their melting, often under a high-purity argon atmosphere. Its advantages include the production of alloys with the desired uniform composition, precise control of the melting process during production, and high-purity samples formed.^104,105^ However, its limitation is the melting and joining of metals with large differences in melting point and specific gravity.^106^ Studies have reported remarkable tribocorrosive behavior and low elastic modulus (∼65 GPa) with this production technique.^5,23^ On the other hand, cold crucible levitation melting (CCLM) uses an induction furnace and allows the melting of metals with a high melting point, favoring uniform and contamination-free alloys due to the molten metals being levitated in the crucible. CCLM can melt metals quickly, as well as agitate the melt while it is in a state of levitation. In addition, by feeding the coils with alternating current, currents are generated in both the crucible and the metals. The heat from these currents melts the metal, while an electromagnetic repulsion force is created. When this force exceeds gravity, the metal is suspended from the crucible and remains suspended in the air. Thus, alloys of uniform solid composition can be created.^34,106,107^

#### 3.3.2 Postprocessing Techniques

For a metallic material to be widely used as an implant, promising biological requirements for osseointegration are desired and necessary. Therefore, regardless of the processing method, be it casting, melting, or sintering, to make the surfaces bioactive, and establish a desired microstructure and advantageous properties, postprocessing is used to acquire the so-called ideal surface. When additive manufacturing is used to produce materials, the layer-by-layer method does not guarantee uniformity and surface quality.^97^ The difficulties inherent in the process, such as lack of fusion, powder agglomeration, cracks, thermal stress, and porosity formation, significantly impact the microstructure of materials, affecting their physical, chemical, and mechanical properties, and final biological performance.^108,109^ Therefore, it is crucial to implement postprocessing to enhance the surface and adjust the biomechanical performance, microstructure, and properties of the materials.^48^ Techniques such as stress relieving, annealing, and aging are commonly employed to increase the stability of the β phase in Ti alloys, especially for specific biomedical applications.^48^ For alloys obtained by subtractive methods, it is important to carry out postprocessing that improves the elastic performance, promotes ultrafine granulations, and enables the manipulation of the composition, thus guaranteeing a refined and homogeneous microstructure. For example, the alloy Ti-35Nb-2Ta-3Zr obtained by casting, after being subjected to friction and agitation processes, showed a reduction in the modulus of elasticity and hardness.^110^ These adjusted mechanical properties are particularly desirable for biomedical applications. The choice of the appropriate postprocessing method depends on the properties required, such as corrosion resistance, hardness, biocompatibility, and desired mechanical properties. Postprocessing can be categorized as physical, chemical, and biological, each playing a distinct role in optimizing the properties of β-type Ti alloys, as discussed below.

##### 3.3.2.1 Physical

Physical methods such as abrupt temperature variations allow adjustments to be made to the hardness, mechanical strength, and microscopic structure of materials.^27,34^ Aging involves heating the alloy to an intermediate temperature for a specific period, followed by controlled cooling. Annealing, common after forming processes, involves heating the alloy to reorganize the atoms followed by rapid cooling.^48,111^ These treatments facilitate the formation of fine, uniformly dispersed precipitates in the alloy matrix, promoting crystalline recovery, reduction of internal defects, and improvements in mechanical properties and corrosion resistance.^27,34,108^ Postprocessing heat treatment (PSHT), for example, allows phase composition to be adjusted, residual stresses to be relieved and the microstructure to be modified through controlled heating and cooling cycles.^108^

In titanium alloys, thermal cycles refine the morphology and distribution of α particles in the β matrix,^112^ improving ductility, eliminating brittle phases, and refining grains. Raising the temperature decomposes the α′ phase into an α+β phase, resulting in coarser β grains that increase ductility but reduce mechanical strength.^108,112^ Ozan et al., 2019^113^ applied heat treatments to the Ti-40.7Zr-24.8Nb alloy, observing greater microhardness, elongation, and toughness after cold rolling and recrystallization, due to finer grains and reduced density. In addition, Mohammed et al., 2015^114^ showed that the modulus of elasticity of the Ti-20.6Nb-13.6Zr-0.5V alloy decreases with increasing cooling rate, and treatments at temperatures above the β transition promote the complete dissolution of the α phase and transformation to the β phase.

Hot isostatic pressing (HIP), which combines high pressure with high temperatures, is indicated to reduce internal porosity and homogenize the microstructure.^63,108^ Pressure helps to reduce pores, while heat treatment aims to reduce residual stress, resulting in a more stable martensite with better tensile strength.^63,108^ Interpass cooling is used to refine equiaxed β grains on a micrometer scale and reduce the anisotropy of the microstructure.^108^ Similarly, Shot Peening promotes limited elastic and plastic deformation, refining and orienting the grains for better mechanical strength and reduced anisotropy, as well as precipitating the ω phase.^33,108,111^ In addition, mechanical friction treatment (SMAT) refines grains and improves the surface, facilitating effective biomedical applications such as protein adsorption and osteoblast adhesion.^115^

Thus, understanding the phase transition process resulting from these treatments is crucial. In β-Ti alloys, the addition of stabilizing β elements, such as Nb and Ta, ensures the stability of the alloy against disturbances to its internal properties with structural stability, given deformation and heat treatments, facilitating the formation of the desired β phase and protecting it against reductions in hardness, tensile strength, fatigue strength, and brittleness. Consequently, the correlation between microstructure and phase transformation is intrinsically linked to the chemical composition and the heating and cooling routes used.^116^

##### 3.3.2.2 Chemical

Chemical postprocessing uses acids or bases to remove contaminants from the surface, form a protective oxide layer, and influence the release of metal ions in implants, affecting their stability and biocompatibility.^109^ Lario et al., 2018^109^ studied the effect of double acid etching with HF and HCl on a β alloy, Ti-35Nb-10Ta-1.5Fe, observing the formation of a thicker and more stable film due to the high reactivity of Nb and Ta, improving the topography and surface chemical composition. This method had a more significant impact on the β alloy compared to Ti-6Al-4V, highlighting the advantage of these materials.^109^ Choe et al., 2010,^117^ in turn, developed nanotubes followed by hydroxyapatite deposition on β-crystallographic substrates such as Ti-30Nb-15Zr, resulting in more regular nanotubes and a more homogeneous and corrosion-resistant HA film. This chemical surface modification provides nanoscale bone reinforcement by the nanotubes and promotes better cell adhesion and proliferation by the HA coating.^117^

##### 3.3.2.3 Biological

The implementation of postprocessing to improve the biological effectiveness of materials, regardless of the surface manufacturing method, focuses on creating bioactive layers that facilitate cell proliferation and offer additional antimicrobial properties.^118^ Chemical processing is crucial in this context, integrating elements with osteoinductive and antimicrobial capabilities that promote osseointegration through bioactive layers.^61^ Especially in β-Ti alloys, adding elements with low cytotoxicity favors passivation and improves corrosion resistance, contributing to better cell compatibility.

Complementing physical methods, these approaches establish ideal surface conditions for biomedical applications. Liu et al., 2023^119^ analyzed the Ti-10Mo-6Zr-4Sn-3Nb alloy and observed, through in vitro and in vivo analyses, its nontoxicity and ability to increase MC3T3-E1 cells. In addition, the alloy showed greater adhesion and differentiation of osteoblasts, evidenced by increased secretion of alkaline phosphatase (ALP) compared to the Ti-6Al-4V alloy. These results indicate that this combination of elements offers safe cytocompatibility and potential for osseointegration. In addition to the incorporation of elements, chemical modifications through different surface treatments, such as acid-etched (SLA), microarc oxidation (MAO), and anodic oxidation (AO), can favor and become potentially biological postprocessing.^120^

When analyzing the potential of the Ti-24Nb-4Zr-8Sn alloy treated by MAO, AO, and SLA, Zhang et al., 2020^121^ observed hydrophilic surfaces with different levels of roughness and capacity to promote bone formation, with anodization treatment (AO) being the most advantageous. Elements such as Ti, Zr, Nb, Ru, Ta, Au, Mo, and Sn are safe because they have high biocompatibility and should be investigated further for their effectiveness in implants.^1^ Previous studies point to the promising effects of Nb and the formation of a promising film on the surface, which facilitates bone integration, as does Sn.^120,122^ This variety of modifications, compositions, and treatments of the β-type alloy promotes the manufacture of highly biocompatible devices, which in addition to adequate mechanical properties, characterized by a low modulus of elasticity and high mechanical strength, have an eminent capacity for safe and favorable application in the biomedical scenario.

#### 3.3.3 Relationship between Porosity, Treatment and Modification Methods

Developing surfaces that enable advanced peri-implant bone tissue formation and a strong and stable bone-implant contact for initial functionalization is the subject of studies and material development.^123−125^ The formation and design of a porous structure that provides increased and facilitated anchorage may be the key to successful osseointegration by acting as functionalizing channels for osteoinduction and osteoconduction.^126^ The interconnected porosity conformation of implants is discussed as being beneficial, allowing for bone growth, migration, and cell proliferation, given the increase in surface area, and better oxygen and nutrient supply.^123,126,127^ Acquiring this surface condition is possible by adjusting parameters using techniques such as additive manufacturing, powder metallurgy, or surface treatments, which make it possible to plan different degrees of porosity and the distribution of pores in the biomaterial, in such a way that it resembles a trabecular bone structure, for example, considering the environment in which this device will be installed and respecting the maintenance of its physical and mechanical characteristics.^126,127^

**Figure 4:**
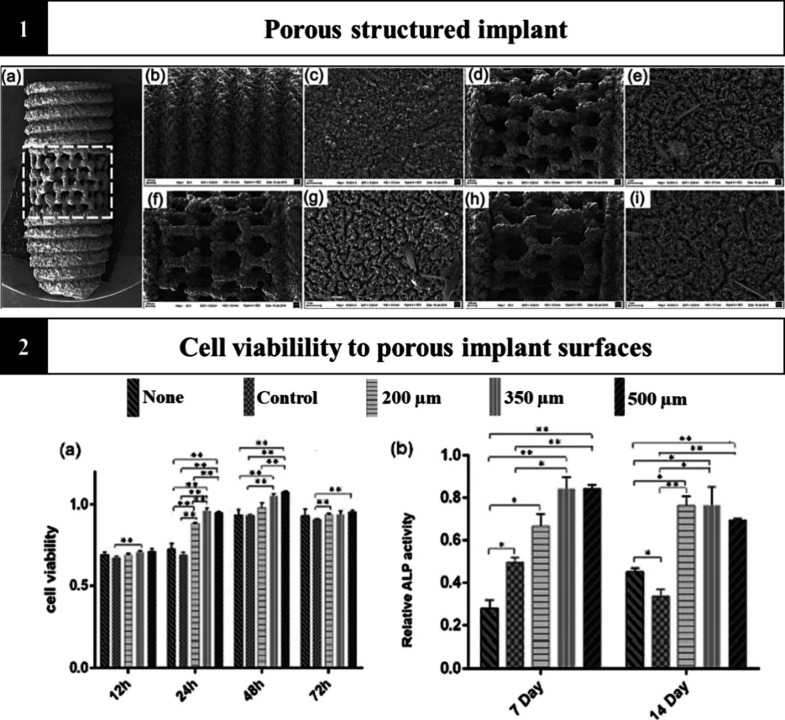
Porous architecture of implants made possible by additive manufacturing favoring cell viability. (1a) Design of a porous implant made by additive manufacturing. (1b) and (1c) refer to the screw-type implant and in terms of the size of the designed pores, (1d) and (1e) are 200 μm pores, (1f) and (1g) are 350 μm pores and (1h) and (1i) are 500 μm pores. (2a) Cell viability and (2b) ALP activity of MC3T3-E1 cells of each group at different time points, whereby *P < 0.05; **P < 0.01. Reproduced or adapted with permission from [^123^]. Copyright [2017] [Nature].

In this promising scenario, one question still needs to be answered: what is the ideal pore size and distribution? Figure 4 (1) shows the formation of an implant with a porous surface with different pore sizes using Laser Beam Melting (LBM) for additive manufacturing. Achieving a porous topography with homogeneous pores is still a limitation and pore size is a decisive factor in bioactivity during tissue regeneration, in which it is described that pores ranging from 150 to 1000 μm can facilitate bone growth.^123−125^ Yang et al., 2017^123^ reported a significant increase in MC3T3-E1 cell viability in implants structured with 350 and 500 μm pores at 24 and 48 h (Figure 4 (2a)). They maintained promising osteogenic activity for up to 14 days. Furthermore, when assessing alkaline phosphatase (ALP) expression levels (Figure 4 (2b)), these increased significantly compared to the control. When osteogenic cells come into intimate contact with the porous surface, they transition from filopodia to lamellipodia, which is essential in this direct interaction with adhesion and proliferation so that the amount of bone matrix deposited is greater and significant.^126^

**Figure 5:**
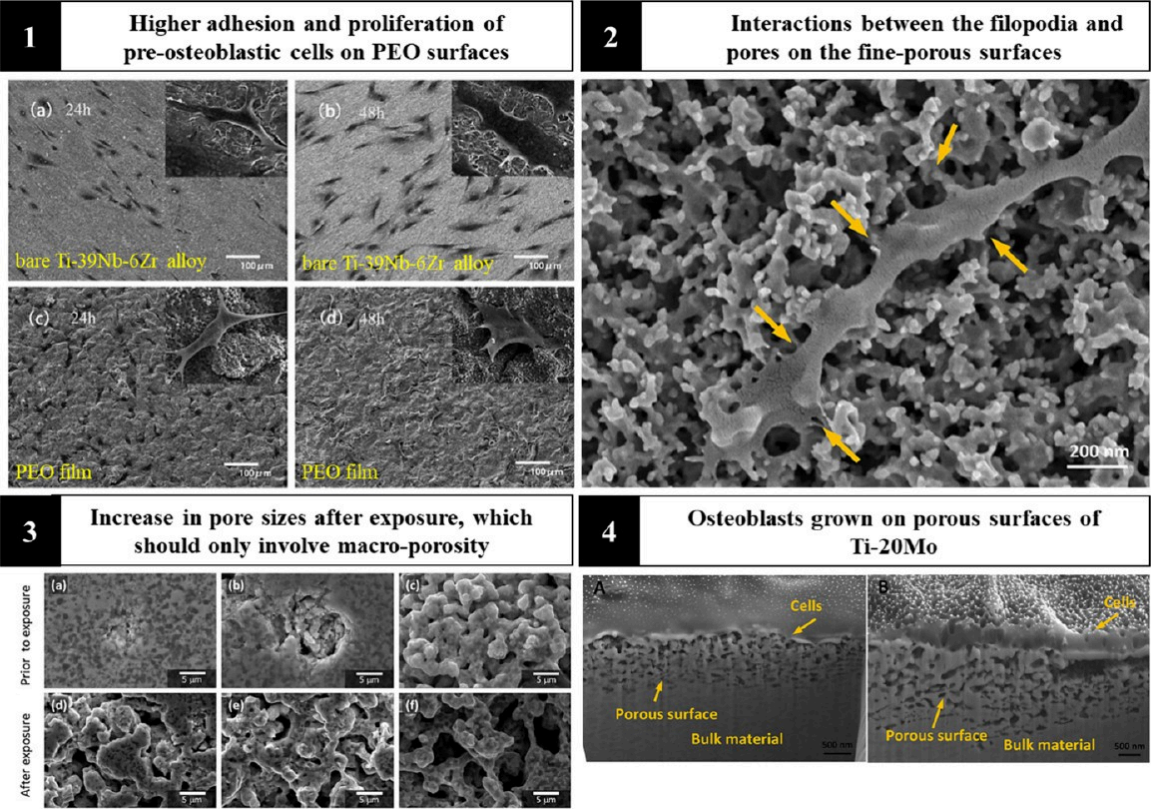
PEO film benefits the proliferation and differentiation of osteoblastic cells and, with its enhanced physical and chemical properties, allows for intimate cell adhesion. Reproduced or adapted with permission from [^127, 128^]. Copyright [2020, 2018] [Elsevier]. Reproduced or adapted with permission from [^49^]. Copyright [2018] [Wiley-VCH].

In addition to this pro-osteogenic advantage of porous topography, a direct relationship can be confirmed between the reduction in modulus of elasticity and the formation of planned porosity in biomaterials, a factor of great importance when it comes to the mechanical failure of devices due to bone stress and consequent resorption.^127^ However, the increase in these recesses and their exposure to the environment can also facilitate corrosive processes by releasing metal ions into the environment, with altered corrosion potential (E_corr_) and corrosion current density (I_corr_) given the lack of passivation within the pores due to the difficulty of oxygen diffusion through them, a limitation to be overcome.^127,128^ Guerra et al., 2020^127^ reported differences in the degree of porosity when simulating exposure to simulated body fluid (SBF) with the Ti-20Nb-11Ta-16Fe-1Mn (at.%) alloy, manufactured by powder metallurgy, and observed an increase in macroporosity, the formation of a biphasic profile of the material with α and β phases and little ion release (Figure 5 (3)). Although the increase in porosity favors a greater surface area and the material may suffer a decrease in corrosion resistance, the number of ions found in the solutions was relatively low, demonstrating that this topographic constitution with added porosity is a promising alternative for bone replacement.^127^

To combine the advantages of low modulus of elasticity, Kuroda et al., 2024^129^ used Ti-15Nb alloy treated with calcium, magnesium, and phosphorus electrolytes. They reported that a porous and homogeneous surface was obtained, discussing the planning of the application parameters, such as the voltage responsible for the size of the pore formed. As well as pore size, voltage influences coating morphology, thickness, crystalline composition, and hardness.^118^ In a directly proportional relationship, the increase in voltage generates an increase in electrical arcs and, with this, the boiling of the electrolyte leads to the formation of numerous oxygen bubbles in the solution and the combination of these factors results in larger pores.^129^ In addition, the higher the intensity of the current applied during the process, the higher the growth rate of the coating on the surface.^129^ An important factor is that the films formed are well adhered, without cracks, and with a more regular surface, with a balance in the formation of the anatase and rutile phases.

Chen et al., 2018,^128^ when coating a β-Ti alloy, Ti-39Nb-6Zr, observed the formation of a more compact coating layer internally and a looser layer externally, but with good adhesion to the substrate. The coating provided greater resistance to corrosion of the alloy and a better coefficient of friction, where the wear debris filled the pores and promoted a surface with relatively smooth areas. In addition, the proliferation of MC3T3-E1 cells was also favored by the coating and its physical and chemical properties. The greater roughness influenced by the processing time favors the distribution of proteins, and the grooves in the coating act as reservoirs for the nucleation and aggregation of proteins, with an efficient and rapid interaction between the cells and the adsorbed proteins.^49,118^ This topographical heterogeneity will be responsible for the initial adhesion of the cells, which in direct contact tend to spread gradually followed by proliferation and differentiation.^128^ As shown in Figure 5 (1), filopodia can attach prominently to the coated surface, whereas this connection is not as strong on the uncoated alloy. At higher magnification, this close interaction between filopodia and material is represented in Figure 5 (2), and a cross-section of the cell-surface interface is confirmed (Figure 5 (4)).

The bioactivity of titanium and its alloys is strongly influenced by their elemental constituents, as well as their complete microstructure, phases, morphology, grain size, and the chemical properties of the outer layers.^129,130^ Therefore, adjustments to the manufacturing process and modifications to the surface play crucial roles in determining biocompatibility and are essential tools for promoting fast and effective osseointegration, as well as offering additional protection against corrosion and improving tribological properties.^129^ Modifying processing parameters and creating bioactive coatings with thick, well-adhered films and porous surfaces are promising approaches. Controlled porosity makes it possible not only to increase the surface area available for bioactive interactions but also to facilitate the diffusion of nutrients and oxygen essential for bone growth and cell proliferation.^129^ This strategy involves not only optimizing porosity but also creating complex, multifaceted geometries that enhance the mechanical and biological properties of titanium implants.^129^

### 3.4 Ensuring Suitability: Key Properties of β-Type Ti Alloys for Dental Implants Application

Ensuring the suitability of materials for dental implant applications is crucial for successful treatments. In this context, understanding the key properties of β-type Ti alloys and their relevance to dental implant applications is essential for clinicians and researchers striving to enhance the performance and durability of implant materials in future clinical practice.

#### 3.4.1 Mechanical and Physical Properties

In the realm of dental implants, meticulous attention to their physical and mechanical properties is paramount. This encompasses factors such as elastic deformation, fatigue resistance, and tensile and compressive strength, all crucial for withstanding diverse loads, particularly those generated during mastication. Consequently, it is essential to ensure compatibility with surrounding bone tissues to mitigate risks of fatigue-induced failures or fractures.^48,113^ In this context, the Ti-6Al-4V alloy is widely used being recognized for its high tensile strength (170–1100 MPa) and yield strength (240–1170 MPa), with a density of 4.5 g/cm^3^ that provides an excellent strength-to-weight ratio, ensuring the long-term stability and durability of implants.^48,131,132^ On the other hand, β titanium alloys also have remarkable mechanical properties, with yield strengths ranging from 367 to 1077 MPa and tensile strengths from 622 to 1143 MPa, as well as a comparatively lower density compared to Ti-6Al-4V.^133−135^ These characteristics allow for equally significant flexibility in implant design and make it easier to adapt the mechanical properties to the needs of the surrounding bone. For example, Saood et al., 2021^136^ demonstrated that the compressive strength of Ti-7Mo-8Nb was 1771 MPa, while that of Ti-6Al-4V was 1738 MPa, indicating the mechanical superiority of Ti-7Mo-8Nb. This is supported by the measured hardness of 380 HV for Ti-7Mo-8Nb, compared to 307 HV for Ti-6Al-4V.^136^ Overall, the key to mechanical success is to achieve an optimal implant design that strikes a balance between a low elastic modulus, comparable to bone, and high strength, facilitating sustained loading over time and reducing the need for surgical interventions.^137−139^

**Figure 6:**
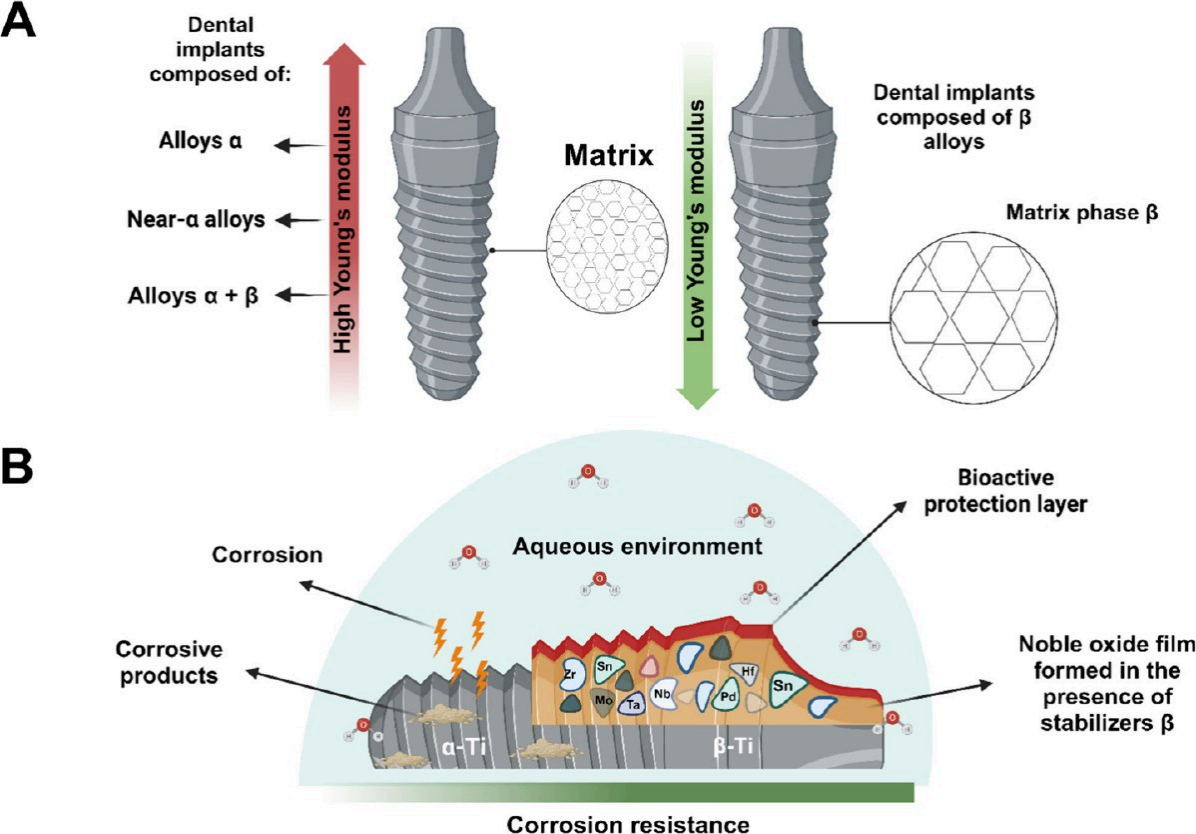
Schematic representation depicting the elastic modulus and corrosion resistance of β-type Ti alloys. (A) Illustration comparing the elastic modulus to phases of the microstructural matrix, demonstrating lower modulus values for implants manufactured from β-type alloys. (B) Comparative illustration showcasing the corrosion process on Ti surfaces within different matrix phases, featuring α-alloy (e.g., cpTi) and β-alloy. The presence of constituent elements in β-type Ti alloys is understood to passivate and generate noble films on the biomaterial surface, providing additional protection against corrosive processes in aqueous environments. The figure was created with BioRender.com (License number: IM26JQ5RZY).

In this context, Ti alloys in the β phase are recognized for their lower elastic modulus compared to α types or biphasic alloys (Figure 6A).^89^ It is proposed that incorporating elements such as Nb, Mo, Hf, or Ta may fortify the strength and lower the modulus of Ti within the body-centered cubic arrangement of these alloys, consequently mitigating implant toxicity. This is attributed to the structural transformation whereby the elasticity modulus of titanium is modulated by the characteristics of the introduced elements, culminating in an overall reduction in the alloy’s elasticity modulus.^140^ Moreover, the higher concentration of these added elements effectively suppresses the formation of the ω phase, thereby reducing the elastic modulus in Ti alloys. This occurs by lowering the transformation temperature, which in turn shifts the phase transition toward the boundary between (β + α′)/β as the temperature approaches room temperature, thereby resulting in a decrease in the elastic modulus by softening the crystal lattice.^141^

Although it is known that the strategy for improving mechanical compatibility is associated with the presence of β-stabilizing elements, it is worth noting that their absence leads to the formation of α′ martensitic transformation and consequent double hardening, resulting in a marked deterioration in the yield strength of the material. On the other hand, its excess increases tensile strength.^69,83^ The hardness of β-type Ti alloys is generally attributed to their more robust solid solution, in contrast to the martensitic phases. However, it is important to note that the β phase itself has higher hardness. This phenomenon has been observed in alloys such as Ti-25Ta-20Zr, Ti-25Ta-30Zr, and Ti-25Ta-40Zr, where an increase in hardness was noted, attributed to the presence of the β phase. This increase can be further attributed to the inclusion of the α′ phase, which acts as an obstacle to the movement of dislocations within the material.^60,68^ Additionally, by increasing the concentration of Zr in the alloy, there is a corresponding increase in the percentage of the β phase. It is noteworthy that the β phase, having the lowest elastic modulus among the phases in Ti alloys, contributes to a decrease in the elastic modulus.^68^

Another noteworthy factor in reducing Young’s modulus of β-type Ti alloys is the decrease in the electron-atom ratio (e/a), representing the average number of valence electrons per atom in the free atom configuration. Such development would involve precise control over the crystalline orientation and the formulation and concentration of β-phase elements with a low ratio.^73,142^ For instance, Young’s modulus of the Ti-15Mo-5Zr-3Al alloy exhibits significant variation across different crystallographic directions, reaching as low as 44.4 GPa along the⟨100⟩ direction, close to human cortical bone.^73,125^ Similarly, a single crystal of β-type low modulus Ti-Nb-Ta-Zr alloy oriented in the ⟨100⟩ direction displays a reduced Young’s modulus (35 GPa) compared to orientations such as ⟨111⟩ and ⟨110⟩, where the modulus aligns with the upper range for bone (approximately 30 GPa).^143^

Regarding variations in concentration, the Ti-11Nb-3.5Fe alloy, characterized by a higher presence of the β phase, exhibits notable ductility and strength, resulting in improved tensile strength and greater plastic deformation capacity compared to the Ti-11Nb-0.5Fe alloy.^48^ Moreover, metastable multicomponent β-type Ti alloys offer a lower Young’s modulus compared to corresponding binary systems with similar thermodynamic stability, attributed to a broadening of β-type Ti alloys electronic hybridization shifting from covalent to more metallic bond states.^139,142^ This transition is facilitated by the body-centered cubic structure, which reduces anisotropy in plastic deformation compared to α alloys, where competition between various slip modes occurs.^139,142^

Overall, the advancement of β-type Ti alloys has led to the creation of novel compositions such as Ti-15Mo-1 Bi, offering a desirable combination of high strength and low modulus,^144^ and Ti-13Nb-13Zr, which closely matches the elastic modulus of human bone.^145^ Moreover, porous Ti-24Nb-4Zr-8Sn samples have been shown to exhibit superior fatigue resistance due to their superelastic property and reduced Young’s modulus, making them promising candidates for biomedical applications requiring compatibility and mechanical properties close to natural bone.^86^

#### 3.4.2 Electrochemical and Tribocorrosive Properties

In addition to mechanical properties, corrosion resistance is also paramount in determining the durability of dental implants. This is because insufficient resistance can result in the release of metal ions and incompatible metalloprotein complexes into tissues, causing allergic and toxic reactions. Consequently, biomedical materials must exhibit high corrosion resistance to ensure longevity.^84,146^ Particularly in dental implants, tribocorrosion exacerbates corrosion processes as wear particles generated during mechanical wear interact with the body’s environment, intensifying corrosion phenomena.^5^ Additionally, debris formation from wear and tribocorrosion processes can lead to bone loss around the implant, characterized by decreased bone-implant fixation, tissue inflammation, toxicity, and potentially cancer development.^5,147,148^

Among biomedical materials, the quest for corrosion-resistant alloys with superior biocompatibility and mechanical properties has led to a growing interest in β-type Ti alloys. This promise originates from the inherent resistance demonstrated by the elements commonly employed in these alloys, particularly in physiological fluids, where they develop a protective noble oxide film (Figure 6B). This film acts as an effective barrier against corrosive ions and ion release, playing a crucial role not only in corrosion protection but also in tissue compatibility.^70,82,149^ The stabilizing agents present in these alloys play a pivotal role in controlling their corrosion behavior. For instance, studies have revealed that Nb positively influences passivation behavior by reducing anion voids in the oxide layer, primarily composed of Ti and Nb oxides.^67,86^ Similarly, titanium alloys containing Mo exhibit superior corrosion resistance, as demonstrated in anodic polarization studies, where Ti-15Mo outperformed commercially pure titanium (cpTi grade IV), Ti-6Al-4V, and Ti-6Al-7Nb in corrosive environments. This suggests Ti-15Mo-1 Bi alloys release fewer metal ions than Ti-6Al-4V.^145^

It is also important to note that changes in the structure of the oxide film or variations in its ionic or electrical conductivity can affect the resistance of the passive film to corrosion. In a study carried out by Martins Junior et al., 2018,^65^ for example, none of the passive films formed on the different alloys tested showed a rupture potential in the range investigated, indicating a high resistance in the test solution used. Similarly, Kumar et al.,2016^88^ demonstrated that the Ti-29Nb-13Ta-4.6Zr alloy showed the lowest corrosion rate, providing remarkable corrosion resistance compared to other alloys. This behavior is attributed to the presence of rare earth elements and refractories added to the alloy, which confer greater corrosion resistance.^67,88^ It is also suggested that the β-type Ti alloys may undergo a transition from a resistive stage to a reactive stage as the frequency decreases, then returns to the resistive stage. This can be attributed to the formation of a thin, adherent passive film of corrosion products, together with an oxidation layer on the surface of the alloy, which prevents further reactions and consequently increases the resistance to these reactions.^84,86,87^ In addition, it is reported by Qin et al., 2018^150^ that the corrosion behavior of Ti alloys is also influenced by the formation of various phase constituents in their microstructure.^150^ As observed by Ge et al., 2024^151^ during corrosion, the presence of the second phase at the interface with the matrix promotes the preferential formation of pits, accelerating the corrosion of the matrix, which reduces the corrosion resistance of the β alloy evaluated compared to the pure β stabilizer.^151^

Within the materials fabricated of β-type Ti alloys, the phenomenon of self-passivation emerges prominently, characterized by the formation of a robust passive film facilitated by essential alloying elements like Nb and Ta. These elements, through their respective passive oxides (e.g., Nb_2_O_5_, ZrO_2_, and Ta_2_O_5_), contribute significantly to the formation of a protective film, resulting in low corrosion rates and minimal ion release, thereby ensuring minimal toxicity.^5,70,87^ Additionally, it is important to note that the inclusion of Zr serves as a neutral stabilizer, enhancing alloy hardness and mechanical, corrosion, and biocompatibility properties.^5,70^ ZrO, present in both the natural layer and grain boundaries, forms a more stable protective layer, reducing corrosion current density and enhancing resistance.^5^ Research suggests that Zr concentrations up to 40% by weight positively impact properties such as microhardness, corrosion potential, and corrosion current.^5,152^

Electrochemical corrosion studies carried out at different immersion times in environments with pH variations, such as Ringer’s solutions, fluorinated saliva, and physiological saline solution, corroborate the influence of these conditions on the corrosion resistance of β-titanium alloys.^153^ Despite the favorable electrochemical properties of β-type Ti alloys, their susceptibility to degradation in oral environments due to constant exposure to factors such as saliva, pH variations, biofilm, and fluoride cannot be overlooked.^4,154^ Over time, these corrosive factors, coupled with wear from implantation procedures, can release metal ions and particles from the implant into surrounding tissues, posing risks of inflammatory reactions and peri-implant bone loss.^14^ In this sense, it was reported by Boraei et al., 2023^155^ that shorter periods of immersion in physiological saline solution improve corrosion resistance due to the formation of internal and external layers of protective noble oxide. In addition, Elshamy et al., 2023^156^ observed that the corrosion resistance of the 70Ti-20Zr-7.5Nb-2.5Ta alloy increases with immersion time, both with and without fluoride ions. These findings are consistent with the results of Boraei et al., 2023,^157^ who highlight the significant influence of immersion in fluids, including pH variations, on the corrosion resistance of β-titanium alloys. They demonstrated that, in the absence of immersion, the corrosion resistance of the β-titanium alloy is greater than when there is immersion or a reduction in immersion time. In addition, it was revealed that in acidic conditions, the corrosion resistance of this same alloy is reduced.^156^

**Figure 7:**
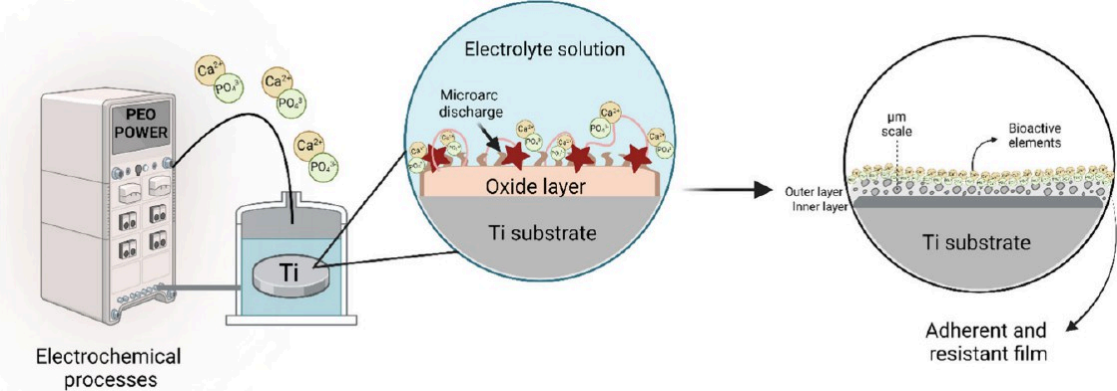
Representative illustration of the PEO process, which allows the formation of a porous film with excellent adhesion to the titanium substrate and the creation of an oxide layer enriched with bioactive elements. Created with BioRender.com (License number: HT272VEBGW).

Consequently, surface treatments have been explored to enhance the bioactivity and corrosion resistance of these materials.^14,154^ These techniques aim to minimize corrosion damage, prevent the penetration of corrosive products, and inhibit electrochemical reactions in the underlying metal to ensure the longevity and optimal performance of dental implants, thus reducing the likelihood of serious biological complications.^14,154^ The heat treatment described by Wang et al., 2019,^158^ improved the corrosion resistance of Ti-35Nb samples by facilitating the homogeneous formation of titanium and niobium oxides due to the greater chemical homogeneity achieved,^158^ as well as laser treatment that promotes grain size refinement and increases the density of its contour, resulting in a lower corrosion current in the dynamic potential.^159^ The plasma electrolytic oxidation (PEO) stands out for its effectiveness,^14,154^ as shown in Figure 7. This electrochemical process creates a porous film that is resistant and adherent to the substrate, enabling the incorporation of bioactive elements like calcium (Ca) and phosphorus (P), crucial for promoting osseointegration.^14,154^ According to Cordeiro et al., 2019,^15^ PEO can significantly improve electrochemical parameters, indicating enhanced stability and passivity, with a thicker double layer (dense and porous), attributed to the formation of an oxide layer that facilitates complete oxidation of alloying elements, contributing to superior corrosion resistance.^160,161^

#### 3.4.3 Cytocompatibility and Antimicrobial Properties

Given the discussed enhancements in mechanical and electrochemical properties of β-type Ti alloys, further exploration into their biological characteristics is paramount to comprehensively address the challenges associated with dental implant applications. Ti alloys have long been favored for use in implantable devices due to their corrosion resistance and biocompatibility, yet ongoing studies continue to shed light on their efficacy. This discussion emphasizes two primary aspects: first, the potential release of titanium into surrounding tissues, which can elicit a spectrum of reactions from mild to severe, including inflammation and implant loosening. Second, the absence of alloying element particles such as Al and V in β-type Ti alloys suggests that they may offer superior biocompatibility,^67,146^ due to the elimination of potentially harmful alloying components and the introduction of low-toxicity elements such as Mo, Sn, Ta, and Nb.^61^ However, it is also important to emphasize that although β-type Ti alloys are safe due to their biological inertness, most of them are not bioactive, which means that osseointegration may need to be improved, even without the presence of potentially dangerous alloying elements.^67,89^

The initial interactions between cells and materials are influenced by the chemistry and properties of the surface, as well as the type of cells and macromolecules present at the biological interface.^89,146^ Nb-containing β-type Ti alloys promote cell activity and strong cell-substrate interaction, resulting in greater cell adhesion and reduced toxicity.^162^ Similarly, the Ti-15Mo-1 Bi alloy demonstrates a good ability to promote continuous bone growth without subsequent absorption, which highlights its unique characteristics. With a modulus of 80 GPa, lower than that of Ti-6Al-4V, the Ti-15Mo-1 Bi alloy, being less rigid, probably facilitates the bone formation process.^144^ Another example is the Ti-16Nb alloy which has been shown to present excellent biocompatibility, in which studies have revealed its ability to foster a high rate of cell proliferation, with the oxidized Ti-Nb alloy specifically noted for its promotion of osteoblast proliferation. Additionally, porous Ti-Nb has been shown to facilitate the adhesion and growth of bone marrow mesenchymal stem cells in rabbits, all while avoiding any discernible inflammatory reaction.^65^

In exploring the biological outcomes of β-type Ti alloys, one notable example involves the assessment of osteoblast adhesion and proliferation on Ti-Nb-Zr β alloys, offering valuable insights into their biocompatibility and cellular response dynamics. For example, the cell culture findings of Mishchenko et al., 2020^40^ the study demonstrated satisfactory initial osteoblast adhesion to both cpTi and Ti-Nb-Zr β alloys, followed by notable cell proliferation after 3 and 7 days of incubation, indicating excellent biocompatibility and the absence of toxicity.^40^ Results from the live/dead assay showed approximately 96% cell viability on the β alloy, further affirming its biocompatibility. Moreover, the study by Nunes et al., 2017^162^ showed a consistent increase in optical density over time by the MTT assay, with a significant rise observed after 7 days of cell proliferation.^162^ Similarly, bone growth around low-modulus Ti-15Mo-1 Bi alloys surpassed that of Ti-6Al-4V alloys at week 12, with this difference becoming even more pronounced after 26 weeks, likely attributable to the low modulus and other unique characteristics of the alloy.^144^

In addition to biocompatibility, preventing biofilm accumulation on dental implant materials is crucial for successful treatment outcomes.^74,162^ In dentistry, peri-implantitis poses a significant threat, potentially leading to the loosening or complete loss of dental implants due to bacterial infections. To address this challenge, β-type Ti alloys with long-lasting antibacterial properties have been developed by incorporating antibacterial elements such as copper (Cu), silver (Ag), and gold (Au), known for their antibacterial and stable activities.^87^ These alloys offer promising solutions for combating biofilm formation and enhancing the longevity of dental implants.

Among antibacterial β-type Ti alloys, copper (Cu) stands out as a preferred antibacterial agent compared to silver (Ag) and gold (Au) due to its low toxicity, high cell compatibility, and remarkable antibacterial properties.^163^ Notably, the Ti-6Al-4V-Cu sample demonstrates superior antibacterial resistance, attributed to the presence of copper in its composition, which releases ions in sufficient quantities to eliminate bacteria and impede their growth.^87^ Recent findings by Yi et al., 2021^163^ support this notion, as colonies of *Escherichia coli* and *Staphylococcus aureus* were observed in the sample with 4% copper by weight, while only a small number of bacteria were detected in the sample with 7% copper by weight. Moreover, almost no bacterial colonies were observed in samples containing 10% and 13% copper by weight, indicating a substantial enhancement in antibacterial properties with increased copper content. This improvement is attributed to copper’s ability to modify protein structure and function or induce degradation using superoxide radicals, resulting in a “multifaceted” impact on the target site.^163^ Nevertheless, achieving the delicate balance between antimicrobial efficacy and cytotoxicity represents a major challenge in the development of titanium alloys for implantable devices.^74,162^ Additionally, studies that evaluate all key properties of materials of β-type Ti alloys, ranging from their mechanical and electrochemical characteristics to their biological interactions, are scarce. Thus, the effect of β-type Ti alloys on mechanical properties and biological and microbiological responses needs to be analyzed by further studies.

## 4 β-Type Ti Alloys for Implant Application: An Evidence-Based Review

Through a systematic search of studies investigating the mechanical and electrochemical properties of β-type Ti alloys, we can deepen our understanding of their suitability and potential in biomedical contexts. Thus, this section offers a comprehensive overview of the existing literature concerning the utilization of β-type Ti alloys in implant applications not only synthesizing previously mentioned information but also addressing it in a quantitative and discussed way, using the results obtained in the studies included in the systematic search described. To achieve this, an extensive systematic search was conducted across four prominent electronic databases: PubMed, Web of Science, Embase, and Scopus. The focus was on identifying studies that provided well-documented data specifically related to β-type Ti alloys intended for biomedical and dental implant applications. Inclusion criteria prioritized studies that incorporated elastic modulus and/or electrochemical assays, while those involving alloys other than β-type Ti alloys were excluded. Additionally, when the studies included in this review conducted microbiological and/or biological assays, we also collected this data set. The key findings extracted from the included studies were synthesized and presented through graphs and tables, with detailed methodological information and search strategies (Table S1) outlined in the Supplementary Material and Methods section.

### 4.1 Synthesis of Research Evidence

**Figure 8:**
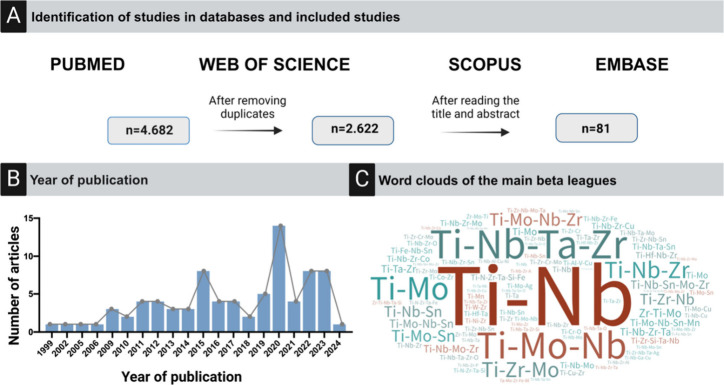
A systematic literature search in electronic databases was conducted to identify studies related to β-type Ti alloys, focusing on elastic modulus and corrosion resistance for implant applications. (A) A comprehensive search in the four main databases - PubMed/MEDLINE, EMBASE, Scopus, and Web of Science - was performed to ensure comprehensive coverage of relevant studies. Following screening, a total of 81 studies were included. (B) A bar graph was generated to visualize the temporal evolution of the studies, with blue bars representing the number of in vitro articles, providing a clear overview of distribution over time. (C) A word cloud was created to highlight the main β-type Ti alloys mentioned in the reviewed studies, allowing for a quick overview of the most relevant alloys in the literature. Created with BioRender.com (License number: OJ276MLRFI).

The initial search retrieved 4,682 records from all searched databases and manual compilation. After removing duplicates, 2,622 records remained for screening based on titles and abstracts. From the 98 articles assessed in full-text, 10^2,84,95,128,164−169^ were excluded as they did not meet the inclusion criteria since the objective was not to evaluate the properties of β-type Ti alloys but rather the influence of different processing routes, as well as 7^116,170−175^ was excluded because they evaluated the influence of different surface treatments and, finally, 1^176^ was excluded due to the lack of evaluation of the elastic modulus and/or electrochemical properties. Thus, 81 (refs (3, 5, 14, 20−23, 30, 40, 42, 45, 65, 68−70, 73, 86, 87, 89−91, 121, 125, 136, 163, and 176−228)) studies were included in this review (Figure 8A). These studies, published over the last 24 years, have revealed a notable increase in the publication rate, with 2020 being the year in which there was a large number of publications on the subject (Figure 8B) and a great diversity of β-type Ti alloys (Figure 8C). However, a relevant point worth to note is the small number of studies evaluating mechanical properties alongside microbiological and biological behavior, in which only 19 studies (refs (65, 68, 69, 121, 163, 176, 180, 182, 184, 187, 196, 198, 200, 204, 207, and 210−213)) incorporated these assays in their study’s experimental design.

### 4.2 Design and Manufacture of β-Type Ti Alloys

As shown in Figure 5C, the alloys most used in the included studies are characterized by binary alloys composed of the elements Ti-Mo and Ti-Nb, followed by ternary alloys such as Ti-Mo-Zr, Ti-Nb-Mo, and Ti-Nb-Zr. In addition, many studies reported here have used quaternary alloys such as Ti-Nb-Zr-Ta.

Regardless of the elemental composition, Nb is the key element associated with titanium for producing β alloys, providing structural reinforcement and enhanced mechanical properties. Mo and Ta, known as β-stabilizers, also play crucial roles in preventing phase transformations, such as the ω phase.^30^ Guo et al., 2015^185^ support this, highlighting that low contents of these elements can lead to undesired phase transitions, such as β to α′. 37.3% of the studies included ternary alloys, highlighting the crucial role of combining elements to enhance the properties and applications of these materials.

Approximately 76% of the studies employed arc melting for casting the alloys. Additionally, about 5% used powder metallurgy, 2% employed cold crucible levitation melting, and another 5% utilized advanced methods such as powder bed fusion, Laser-directed energy deposition, Plasma rotating electrode process, or Vacuum electromagnetism induction furnace, while 12% did not specify the method used. Gebert et al., 2015^229^ elucidate that arc melting is favored due to its high temperature ensuring purity, precise alloy composition, uniform melting, and ability to handle materials with different melting points effectively.

### 4.3 β-Type Ti Alloys: Promising Candidates for Low Elastic Modulus Implants

CpTi and biphasic α + β Ti alloys, such as Ti-6Al-4V, are widely used in implant dentistry due to their well-established physical, chemical, and mechanical properties.^62,77^ However, these materials suffer from a high elastic modulus compared to natural bone, leading to ″stress shielding,″ which can hinder proper implant integration.^71,72^ Cp-Ti and non-β titanium alloys such as Ti-6Al-4V, were commonly used as control groups in evaluating new β-type titanium alloys. Specifically, cpTi was utilized in 11 studies, and Ti-6Al-4V in 18 studies. Cordeiro et al., 2019^14^ demonstrated that incorporating β-stabilizing elements like Nb and Ta into TiNbZrTa alloys reduces their elastic modulus. For example, Ti-35Nb-7Zr-5Ta has an elastic modulus of approximately 82 GPa, compared to around 102 GPa for cpTi, indicating a significant reduction due to the presence of these alloying elements.^14^

Studies consistently show that the presence of β elements in titanium alloys correlates with a reduction in elastic modulus.^86,89,199,230^ Nunes et al., 2023^89^ observed that Young’s modulus decreases with increasing Mo content in Ti-26Nb-xMo alloys (x ranging from 0 to 8% by weight). However, exceptions have been noted, as described by Zhang et al., 2020^231^ where the elastic modulus initially dropped to 69.2 GPa in Ti-10Nb alloy but increased to 87.9 GPa in Ti-15Nb alloy before decreasing again to 77.1 GPa in Ti-25Nb alloy. Despite these variations, all elastic modulus values for β-type titanium alloys remain lower than those of non-β controls.

**Figure 9:**
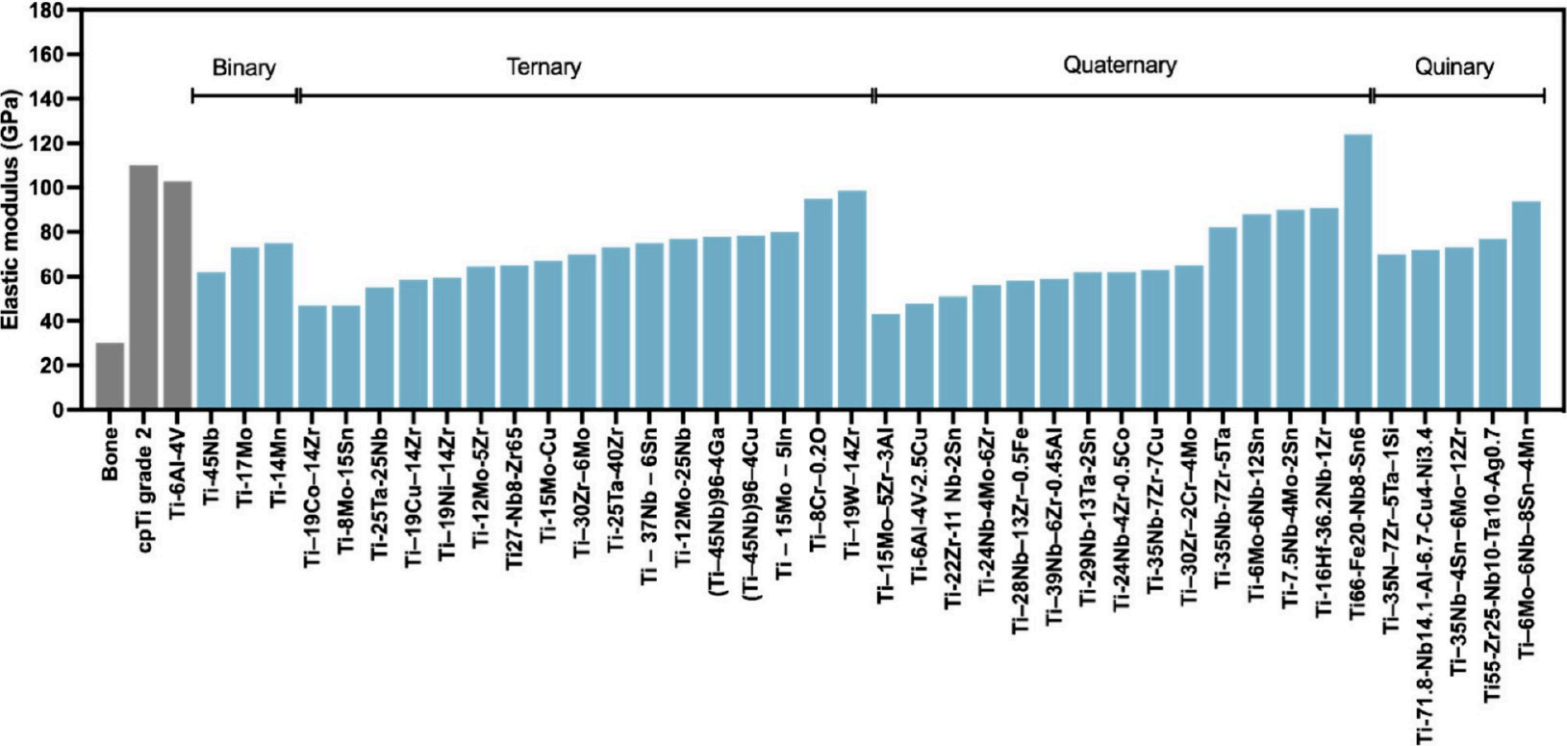
A summary of the elastic modulus of β-type titanium alloys investigated by the included studies. The graph displays the average elastic modulus for each alloy composition. In cases where multiple alloys shared the same composition but varied in weight percentages of each element, the reported value represents one example of those alloys.

The studies reviewed show that the inclusion of β elements in alloys is related to a reduction in the modulus of elasticity. This means that these alloys tend to be more flexible and less rigid. This information is illustrated in Figure 9 and detailed in Table 1, which summarizes the control and experimental groups of the articles reviewed and presents a ranking of the modulus of elasticity values found. However, it is important to consider some limitations and variations in the studies. Limitations can affect the accuracy and generalizability of the results, such as the methodology and types of alloys tested. In addition, variations in results were observed between studies, with significant differences that may result from different experimental conditions or additional factors not fully explored. The predominance of cpTi and Ti-6Al-4V as controls highlight the need to explore a wider range of reference materials, including existing β-type Ti alloys.

**Table 1:** Summary of β-Type Ti Alloys Evaluated in the Included Studies Regarding the Composition, Processing Route, Elastic Modulus, and Corrosion Current Density Results^a^

	**β -type Ti alloy**		**Elastic modulus (GPa)**		
**Author**	**Composition (wt%)**	**Processing route**	**Control**	**β alloy**	**Elastic modulus ranking^b^**
**Afonso et al**. **(2017)**(214)	66Ti-20Fe-8Nb-6Sn	HEBM	63Ti-23Fe-8Nb-6Sn: 127.8	66Ti-20Fe-8Nb-6Sn: 124	66Ti-20Fe-8Nb-6Sn < 63Ti-23Fe-8Nb-6Sn
**Alberta et al**. **(2022)**(215)	(Ti-45Nb)96–4 Ga; (Ti-45Nb)96–4Cu; (Ti-45Nb)96–2 Ga-2Cu	Arc melting	Ti-45Nb: 64.2 ± 2.0	(Ti-45Nb)96–4 Ga: 77.7 ± 3.3; (Ti-45Nb)96–4Cu: 78.5 ± 2.5; (Ti-45Nb)96–2 Ga-2Cu: 73.4 ± 1.9	Ti-45Nb< (Ti-45Nb)96–2 Ga-2Cu< (Ti-45Nb)96–4 Ga< (Ti-45Nb)96–4Cu
**Almeida et al**. **(2020)**(216)	Ti-12Mo-25Nb	Arc melting	cpTi: 100 ± 0,2; Ti-6Al-4V: 118 ± 2	77 ± 0,2	Ti-12Mo-25Nb< cpTi< Ti-6Al-4V
**Arias-González et al**. **(2022)**(213)	Ti-42Nb	Laser-directed energy deposition	Ti-6Al-4V: 110–114	9.4 ± 3.0	Ti-42Nb< Ti-6Al-4V
**Bertrand et al**. **(2010)**(91)	Ti-25Ta-25Nb	Cold crucible semilevitation melting	cpTi: 105	55	Ti-25Ta-25Nb< cpTi
**Brizuela et al**. **(2019)**(230)	Ti-15Zr; Ti-19.1Nb-8.8Zr; Ti-41.2Nb-6.1Zr; Ti-25Hf-25Ta	Arc melting	CpTi: 107; Ti-6Al-4V: 113	Ti-15Zr: 103; Ti-19.1Nb-8.8Zr: 74; Ti-41.2Nb-6.1Zr: 67; Ti-25Hf-25Ta: 53	Ti-25Hf-25Ta< Ti-41.2Nb-6.1Zr< Ti-19.1Nb-8.8Zr< Ti-15Zr< CpTi< Ti-6Al-4V
**Çaha et al**. **(2020)**(218)	Ti-15Nb; Ti-40Nb	NR	Ti-6Al-4V: 112	Ti-15Nb: 88 ± 1; Ti-40Nb: 51 ± 1	Ti-40Nb< Ti-15Nb< Ti-6Al-4V
**Chen et al**. **(2018)**(128)	Ti - 37Nb - 6Sn	Arc melting	NR	≈75	NR
**Cho et al**. **(2014)**(211)	Ti-14Mn	Arc melting	Ti-10Mn: 85	Ti-14Mn: 75	Ti-14Mn< Ti-10Mn
**Cordeiro et al**. **(2019)**(14)	Ti-35Nb-7Zr-5Ta	Arc melting	cpTi: ≈102; Ti-6Al-4V: ≈139	≈82	Ti-35Nb-7Zr-5Ta < cpti < Ti-6Al-4V
**Correa et al**. **(2016)**(219)	Ti-15Zr-7.5Mo; Ti-15Zr-15Mo	Arc melting	cpTi: 105 ± 2	Ti-15Zr-7.5Mo: 114 ± 5; Ti-15Zr-15Mo: 74 ± 4	Ti-15Zr-15Mo< cpTi< Ti-15Zr-7.5Mo
**Cui et al**. **(2009)**(195)	Ti-28Nb-13Zr-0.5Fe	Vacuum electromagnetism induction furnace	NR	≈58	NR
**Dai et al**. **(2013)**(220)	Ti–35Nb–4Sn–6Mo–3Zr; Ti–35Nb–4Sn–6Mo–6Zr;Ti–35Nb–4Sn–6Mo–9Zr; Ti–35Nb–4Sn–6Mo–12Zr; Ti–35Nb–4Sn–6Mo–15Zr	Arc melting	Ti–35Nb–4Sn–6Mo: 82	Ti–35Nb–4Sn–6Mo–3Zr: 92; Ti–35Nb–4Sn–6Mo–6Zr: 85;Ti–35Nb–4Sn–6Mo–9Zr: 69; Ti–35Nb–4Sn–6Mo–12Zr: 73; Ti–35Nb–4Sn–6Mo–15Zr: 75	Ti–35Nb–4Sn–6Mo–9Zr< Ti–35Nb–4Sn–6Mo–12Zr< Ti–35Nb–4Sn–6Mo–15Zr< Ti–35Nb–4Sn–6Mo< Ti–35Nb–4Sn–6Mo–6Zr< Ti–35Nb–4Sn–6Mo–3Zr
**Dai et al**. **(2016)**(196)	Ti-24Nb-4Zr-8Sn	NR	cpTi: ≈140	Ti-24Nb-4Zr-8Sn ≈ 80	Ti-24Nb-4Zr-8Sn < cpTi
**Dang et al**. **(2020)**(197)	Ti-2Zr-0.1Nb-0.1Sn; Ti-2Zr-0.2Nb-0.2Sn; Ti-2Zr-0.3Nb-0.3Sn; Ti-2Zr-0.1Nb-0.1Mo; Ti-2Zr-0.2Nb-0.2Mo; Ti-2Zr-0.3Nb-0.3Mo.	NR	Ti-2Zr: 30.53	Ti-2Zr-0.1Nb-0.1Sn: 14.72; Ti-2Zr-0.2Nb-0.2Sn: 26.47; Ti-2Zr-0.3Nb-0.3Sn: 28.44; Ti-2Zr-0.1Nb-0.1Mo: 29.09; Ti-2Zr-0.2Nb-0.2Mo: 30.44; Ti-2Zr-0.3Nb-0.3Mo: 31.00	Ti-2Zr-0.1Nb-0.1Sn< Ti-2Zr-0.2Nb-0.2Sn< Ti-2Zr-0.3Nb-0.3Sn< Ti-2Zr-0.1Nb-0.1Mo< Ti-2Zr-0.2Nb-0.2Mo< Ti-2Zr< Ti-2Zr-0.3Nb-0.3Mo.
**Estevez et al**. **(2016)**(221)	Ti-19Ta-14Zr; Ti-19Ni-14Zr; Ti-19Cu-14Zr; Ti-19W-14Zr; Ti-19Co-14Zr	NR	Ti-19Mo-14Zr: 90.14	Ti-19Ta-14Zr: 68.99; Ti-19Ni-14Zr: 59.54; Ti-19Cu-14Zr: 58.68; Ti-19W-14Zr: 98.66; Ti-19Co-14Zr: 46.74	Ti-19Co-14Zr< Ti-19Cu-14Zr< Ti-19Ni-14Zr< Ti-19Ta-14Zr< Ti-19Mo-14Zr< ; Ti-19W-14Zr
**Fujisawa et al**. **(2018)**(180)	Ti-Nb-Sn	Arc melting	Ti-Ni-Sn: 78	Ti-Nb-Sn: 45	Ti-Nb-Sn< Ti-Ni-Sn
**Gabriel et al**. **(2017)**(181)	Ti-12Mo-13Nb	Arc melting	Ti-6Al-4V: 140 ± 3.36	110 ± 2.62	Ti-12Mo-13Nb < Ti-6Al-4V
**Godley et al**. **(2006)**(191)	Ti-45Nb	NR	Ti-6Al-4V: 105; Ti-55Ni:80	Ti-45Nb: 62.05	Ti-45Nb< Ti-55Ni < Ti-6Al-4V: 105
**Golasinski et al**. **(2021)**(182)	Ti-36-Nb-2Ta-3Zr0.3O	Powder metallurgy	Ti-6A-l4V: ≈130	≈64.9 - 69.6	Ti-36Nb-2Ta-3Zr-0.3O < Ti-6Al-4V
**González et al**. **(2009)**(183)	Ti-16Hf-36.2Nb-1Zr	Arc melting	Ti-16.2Hf-24.8Nb-1Zr: 74 ± 1.5; Ti-5.2Hf-31.2Nb-0.4Zr: 76 ± 4	Ti-16Hf-36.2Nb-1Zr: 91 ± 2	Ti-16.2Hf-24.8Nb-1Zr< Ti-5.2Hf-31.2Nb-0.4Zr< Ti-16Hf-36.2Nb-1Zr
**Guo et al**. **(2013)**(184)	Ti-35-Nb-2Ta-3Zr	Arc melting	NR	NR	NR
**Guo et al**. **(2015)**(185)	Ti-33Nb-4Sn	Arc melting	NR	≈36	NR
**Hacisalihoglu et al**. **(2015)**(186)	Ti-13Nb-13Zr; Ti-15Mo; Ti-45Nb	Electrode discharge machine (wire-EDM)	cpTi: ≈110; Ti-6Al-4V: ≈114	Ti-13Nb-13Zr: ≈80; Ti-15Mo: ≈100; Ti-45Nb: ≈65	Ti45Nb< Ti13Nb13Zr< Ti15Mo< cpTi< Ti6Al4V
**Hwang et al**. **(2021)**(187)	Ti-39Nb-6Zr-0.45Al	NR	Ti-39Nb-6Zr: 45	59	Ti-39Nb-6Zr< Ti-39Nb-6Zr+0.45Al
**Kopova et al**. **(2016)**(69)	Ti-35N-7Zr-5Ta-1Si; Ti-35N-7Zr-5Ta-2Fe; Ti-35N-7Zr-5Ta-0.5Si-1Fe; Ti-35N-7Zr-5Ta-0.5Si-2Fe; Ti-35N-7Zr-5Ta-1Si-1Fe	Arc melting	Ti-35N-7Zr-5Ta: 65	Ti-35N-7Zr-5Ta-1Si: 70; Ti-35N-7Zr-5Ta-2Fe: 82; Ti-35N-7Zr-5Ta-0.5Si-1Fe: 75; Ti-35N-7Zr-5Ta-0.5Si-2Fe: 85; Ti-35N-7Zr-5Ta-1Si-1Fe: 79	Ti-35N-7Zr-5Ta< Ti-35N-7Zr-5Ta-1Si< Ti-35N-7Zr-5Ta-0.5Si-1Fe< Ti-35N-7Zr-5Ta-1Si-1Fe< Ti-35N-7Zr-5Ta-2Fe< Ti-35N-7Zr-5Ta-0.5Si-2Fe
**Kumar et al**. **(2009)**(209)	Ti-15Mo	NR	NR	NR	NR
**Kumar et al**. **(2011)**(87)	Ti-25-Mo	Arc melting	NR	NR	NR
**Kumar et al**. **(2019)**(210)	Ti-6Al-4V-2.5Cu; Ti-29Nb-13Ta-4.6Zr	NR	Ti-6Al-4V: 115 ± 0.2	Ti-6Al-4V-2.5Cu: 47.8 ± 0.22; Ti-29Nb-13Ta-4.6Zr: 109.08 ± 0.08	Ti-6Al-4V-2.5Cu< Ti-29Nb-13Ta-4.6Zr< Ti-6Al-4V
**Kuroda et al**. **(2020)**(218)	Ti-25Ta-10Zr; Ti-25Ta-20Zr; Ti-25Ta-30Zr; Ti-25Ta-40Zr	Arc melting	Ti-25Ta: 86	Ti-25Ta-10Zr: 82; Ti-25Ta-20Zr: 80; Ti-25Ta-30Zr: 78; Ti-25Ta-40Zr: 73	Ti-25Ta-40Zr< Ti-25Ta-30Zr< Ti-25Ta-20Zr< Ti-25Ta-10Zr< Ti-25Ta
**Laheurte et al**. **(2010)**(190)	Ti-29Nb-6Ta-5Zr	Arc melting	Ti-29Nb-11Ta-5Zr: 50	43	Ti-29Nb-6Ta-5Zr< Ti-29Nb-11Ta-5Zr
**Lee et al**. **(2012)**(73)	Ti-40V; Ti-79V	Arc melting	Ti-40V: 64; Ti-79V: 118	Ti-15Mo-5Zr-3Al: 43	Ti-15Mo-5Zr-3Al< Ti-40V< Ti-79V
**Li et al**. **(2023)**(208)	Ti-8Mo-3Sn; Ti-8Mo-5Sn; Ti-8Mo-7Sn; Ti-8Mo-9Sn; Ti-8Mo-11Sn; Ti-8Mo-13Sn; Ti-8Mo-15Sn	Arc melting	Ti-8Mo: 58.6	Ti-8Mo-3Sn: 66.9; Ti-8Mo-5Sn: 74.8; Ti-8Mo-7Sn: 76.8; Ti-8Mo-9Sn: 68.7; Ti-8Mo-11Sn: 54; Ti-8Mo-13Sn: 50.2; Ti-8Mo-15Sn: 46.8.	Ti-8Mo-15Sn< Ti-8Mo-13Sn< Ti-8Mo-11Sn< Ti-8Mo< Ti-8Mo-3Sn< Ti-8Mo-9Sn< Ti-8Mo-5Sn< Ti-8Mo-7Sn
**Liu et al**. **(2015)**(66)	Ti-8Cr-0.2O; Ti-7Cr-0.2O	Arc melting	Ti-9Cr-0.2O: 85	Ti-8Cr-0.2O: 95; Ti-7Cr-0.2O: 115	Ti-9Cr-0.2O< Ti-8Cr-0.2O< Ti-7Cr-0.2O
**Martins Junior et al**. **(2017)**(65)	Ti-15Mo-5Nb; Ti-15Mo-10Nb; Ti-15Mo-15Nb; Ti-15Mo-20Nb	Arc melting	Ti-15Mo: 93,62 ± 12,28	Ti-15Mo-5Nb: 76,4 ± 14,12 ; Ti-15Mo-10Nb: 78,65 ± 12,29; Ti-15Mo-15Nb: 76,68 ± 10,31; Ti-15Mo-20Nb: 78,8 ± 8,05	Ti-15Mo-5Nb< Ti-15Mo-15Nb< Ti-15Mo-10Nb< Ti-15Mo-20Nb< Ti-15Mo
**Niinomi et al**. **(1999)**(206)	Ti-13Nb-13Zr; Ti-29Nb-13Ta-4.6Zr; Ti-16Nb-13Ta-4Mo; Ti-29Nb-13T; Ti-29Nb-13Ta-4Mo; Ti-29Nb-13Ta-2Sn; Ti-29Nb-13Ta-4.6Sn; Ti-29Nb-13Ta-6Sn.	Arc melting	Ti-6Al-4V ELI: 112	Ti-13Nb-13Zr: 64–77; Ti-29Nb-13Ta-4.6Zr: 65; Ti-16Nb-13Ta4-Mo: 91; Ti-29Nb-13T: 76; Ti-29Nb-13Ta-4Mo: 74; Ti-29Nb-13Ta-2Sn: 62; Ti-29Nb-13Ta-4.6Sn: 66; Ti-29Nb-13Ta-6Sn: 74.	Ti-29Nb-13Ta-2Sn< Ti-29Nb-13Ta-4.6Zr< Ti-29Nb-13Ta-4.6Sn< Ti-29Nb-13Ta-6Sn; Ti-29Nb-13Ta-4Mo< Ti-29Nb-13T< Ti-13Nb-13Zr; Ti-16Nb-13Ta-4Mo; Ti-6Al-4V ELI
**Niinomi et al**. **(2002)**(207)	Ti-29Nb-13Ta-4.6Zr	Arc melting	Ti-6Al-4V ELI: 120	Ti-29Nb-13Ta-4.6Zr: 65	Ti-29Nb-13Ta-4.6Zr< Ti-6Al-4V ELI
**Nnamchia et al**. **(2019)**(86)	Ti-8Mo-6Nb-4Zr; Ti-8Mo-5Nb-3Zr; Ti-8Mo-4Nb-2Zr; Ti-8Mo-4Nb-5Zr.	Arc melting	Ti-6Al-4V: 113	Ti-8Mo-6Nb-4Zr: 72; Ti-8Mo-5Nb-3Zr: 69; Ti-8Mo-4Nb-2Zr: 35.4; Ti-8Mo-4Nb-5Zr: 52.	Ti-8Mo-4Nb-2Zr< Ti-8Mo-4Nb-5Zr< Ti-8Mo-5Nb-3Zr< Ti-8Mo-6Nb-4Zr< Ti-6Al-4V
**Nunes et al**. **(2023)**(89)	Ti-29Nb-2Mo-3Zr; Ti-29Nb-2Mo-6Zr; Ti-24Nb-4Mo-3Zr; Ti-24Nb-4Mo-6Zr	Arc melting	Ti-6Al-4V: 118 ± 1.0	Ti-29Nb-2Mo-3Zr: 64 ± 2.0; Ti-29Nb-2Mo-6Zr: 62 ± 2.0; Ti-24Nb-4Mo-3Zr: 60 ± 1.0; Ti-24Nb-4Mo-6Zr: 56 ± 1.0	Ti-24Nb-4Mo-6Zr< Ti-24Nb-4Mo-3Zr< Ti-29Nb-2Mo-6Zr< Ti-29Nb-2Mo-3Zr< Ti-6Al-4V
**Okulov et al**. **(2013)**(205)	Ti-71.8-Nb14.1-Al-6.7-Cu4-Ni3.4	Arc melting	Ti68.8-Nb13.6-Al6.5-Cu6-Ni5.1: 82 ± 0.5	72 ± 0.5	Ti-71.8-Nb14.1-Al-6.7-Cu4-Ni3.4 < Ti68.8-Nb13.6-Al6.5-Cu6-Ni5.1
**Popa et al**. **(2012)**(70)	Ti-20Nb-10Zr-5Ta	Arc melting	cpTi:105	59	Ti-20Nb-10Zr-5Ta< cpTi
**Romero-Resendiz et al**. **(2023)**(21)	Ti - 15Mo - 5In	Powder metallurgy	NR	≈80	NR
**Santos et al**. **(2023)**(222)	Ti-40Nb	Arc-Melting (AC), Hot Rolling (HR)	cpTi: ≈100	Ti-30Nb-50Zr: 77 AC, 41 HR	Ti-30Nb-50Zr< Ti-40Nb< Ti-27Nb-39Zr< Ti-20Nb-30Zr-13Ta < cpTi
	Ti-27Nb-39Zr (39Zr)			Ti-27Nb-39Zr: 91 AC, 70 HR	
	Ti-30Nb-50Zr (50Zr)			Ti-20Nb-30Zr-13Ta: 83 AC, 72 HR	
	Ti-20Nb-30Zr-13Ta (30Zr)			Ti-40Nb: 75 AC, 69 HR	
**Santos et al**. **(2023)**(232)	Ti-15Nb	Arc-Melting	cpTi: ≈100	Ti-15Nb: 65	Ti-40Nb< TNZT < Ti-15Nb< TNZ-40< TNZ-33 < cpTi
	Ti-40Nb			Ti-40Nb: 54	
	Ti-33Nb-33Zr			TNZ-33: 78	
	Ti-40Nb-40Zr			TNZ-40: 75	
	Ti-35Nb-5Zr-7Ta (TNZT)			TNZT: 64	
**Saood et al**. **(2021)**(136)	Ti-7Mo-8Nb	Powder metallurgy	Ti-6Al-4V: 115	44.9	Ti-7Mo-8Nb < Ti-6Al-4V
**Schaal et al**. **(2023)**(22)	Ti-22Zr-11 Nb-2Sn	Laser Powder Bed Fusion	Ti-6Al-4V ELI: 111	51	Ti-22Zr-11Nb-2Sn< Ti-6Al-4V ELI
**Silva et al**. **(2023)**(193)	Ti-29Nb-13Ta-4.6Zr	NR	Ti-6Al-4V: ≈ 110	≈42	Ti-29Nb-13Ta-4.6Zr< Ti-6Al-4V
**Song et al**. **(2012)**(194)	52Ti-30Nb-18Zr; 46Ti-24Nb-30Zr; 35Ti-15Nb-50Zr; 27Ti-8Nb-65Zr; 18Ti-82Zr	Arc melting	62Ti-38Nb ≈95	52Ti-30Nb-18Zr ≈ 75; 46Ti-24Nb-30Zr ≈ 74; 35Ti-15Nb-50Zr ≈ 73; 27Ti-8Nb-65Zr ≈ 65; 18Ti-82Zr ≈ 85	27Ti-8Nb-65Zr < 35Ti-15Nb-50Zr < 46Ti-24Nb-30Zr < 52Ti-30Nb-18Zr < 18Ti-82Zr < 62Ti-38Nb
**Straský et al**. **(2022)**(23)	Ti-(26–35)Nb-6Ta-7Zr-0.7O; Ti-(20–35)Nb-7Zr-0.7O	Arc melting	Ti-35Nb-6Ta-7Zr: ≈65	Ti-(26–35)Nb-6Ta-7Zr-0.7O: ≈65; Ti-(20–35)Nb-7Zr-0.7O: ≈115	Ti-35Nb-6Ta-7Zr; Ti-(26–35)Nb-6Ta-7Zr-0.7O < Ti-(20–35)Nb-7Zr-0.7O
**Sutowo (2020)**(223)	Ti-6Mo-6Nb-4Sn-4Mn; Ti-6Mo-6Nb-4Sn-8Mn; Ti-6Mo-6Nb-8Sn; Ti-6Mo-6Nb-8Sn-4Mn; Ti-6Mo-6Nb-8Sn-8Mn	Arc melting	Ti-6Mo-6Nb-4Sn: 103	Ti-6Mo-6Nb-4Sn-4Mn: 96; Ti-6Mo-6Nb-4Sn-8Mn:98; Ti-6Mo-6Nb-8Sn: 99; Ti-6Mo-6Nb-8Sn-4Mn:94; Ti-6Mo-6Nb-8Sn-8Mn: 100	Ti-6Mo-6Nb-8Sn-4Mn< Ti-6Mo-6Nb-4Sn-4Mn< Ti-6Mo-6Nb-4Sn-8Mn< Ti-6Mo-6Nb-8Sn-4Mn< Ti-6Mo-6Nb-8Sn
**Sutowo et al**. **(2019)**(224)	Ti-6Mo-6Nb-4Sn; Ti-6Mo-6Nb-8Sn; Ti-6Mo-6Nb-12Sn	Arc melting	Ti-6Mo-6Nb: 104	Ti-6Mo-6Nb-4Sn: 103.5; Ti-6Mo-6Nb-8Sn: 95.9; Ti-6Mo-6Nb-12Sn: 88.0	Ti-6Mo-6Nb-12Sn< Ti-6Mo-6Nb-8Sn< Ti-6Mo-6Nb-4Sn< Ti-6Mo-6Nb
**Utomo et al**. **(2023)**(177)	Ti-30Nb-2Sn; Ti-30Nb-5Sn; Ti-30Nb-8Sn.	Arc melting	Ti-30Nb: 114	Ti-30Nb-2Sn: 120; Ti-30Nb-5Sn: 112; Ti-30Nb-8Sn: 115	Ti-30Nb-5Sn> Ti-30Nb> Ti-30Nb-8Sn> Ti-30Nb-2Sn
**Wei et al**. **(2020)**(225)	Zr-25Ti-2.5Mo; Zr-25Ti-5Mo; Zr-25Ti-7.5Mo; Zr-25Ti-10Mo; Zr-25Ti-12.5Mo	Arc melting	Zr-25Ti: 18.99	Zr-25Ti-2.5Mo: 18.43; Zr-25Ti-5Mo: 17.74; Zr-25Ti-7.5Mo: 24.44; Zr-25Ti-10Mo: 22.89; Zr-25Ti-12.5Mo: 22.54	Zr-25Ti-5Mo< Zr-25Ti-2.5Mo< Zr-25Ti< Zr-25Ti-12.5Mo< Zr-25Ti-10Mo< Zr-25Ti-7.5Mo
**Xie et al**. **(2015)**(90)	Ti-6Mo; Ti-8Mo; Ti-10Mo	Arc melting	Ti-4Mo: 4.32	Ti-6Mo: 3,28; Ti-8Mo: 8,51; Ti-10Mo: 4,87	Ti-6Mo< Ti-4Mo< Ti-10Mo< Ti-8Mo
**Yang et al**. **(2022)**(203)	Ti-35Nb-7Zr-5Ta; Ti-34.9Nb-6.9Zr-4.9Ta-0.5Si; Ti-34.2Nb-6.8Zr-4.8Ta-2.3Si	Plasma rotating electrode process.	Ti-6Al-4V: 112.14 ± 1.69	Ti-35Nb-7Zr-5Ta: 63.59 ± 2.3; Ti-34.9Nb-6.9Zr-4.9Ta-0.5Si: 65.93 ± 3.1; Ti-34.2Nb-6.8Zr-4.8Ta-2.3Si: 78.51 ± 1.75	Ti-35Nb-7Zr-5Ta< Ti-34.9Nb-6.9Zr-4.9Ta-0.5Si< Ti-34.2Nb-6.8Zr-4.8Ta-2.3Si< Ti-6Al-4V
**Yi et al**. **(2021)**(163)	Ti-35Nb-7Zr-7Cu; Ti-35Nb-7Zr-10Cu; Ti-35Nb-7Zr-13Cu	Arc melting	Ti-35Nb-7Zr-4Cu: 57	Ti-35Nb-7Zr-7Cu: 63; Ti-35Nb-7Zr-10Cu: 74; Ti-35Nb-7Zr-13Cu: 79	Ti-35Nb-7Zr-4Cu< Ti-35Nb-7Zr-7Cu< Ti-35Nb-7Zr-10Cu< Ti-35Nb-7Zr-13Cu
**Yılmaz et al**. **(2017)**(202)	Ti-16Nb-2Sn; Ti-16Nb-4Sn	Arc melting	Ti-16Nb: 124	Ti-16Nb-2Sn: 76; Ti-16Nb-4Sn: 92	Ti-16Nb-2Sn < Ti-16Nb-4Sn< Ti-16Nb
**Yılmaz et al**. **(2018)**(227)	Ti-16Nb-15Zr	Arc melting	Ti-16Nb: 110	105	Ti-16Nb-15Zr< Ti-16Nb
**Yuan et al**. **(2022)**(201)	Ti-15Mo-Cu	Arc melting	Ti-15Mo: 79 ± 2	67 ± 3 GPa	Ti-15Mo-Cu< Ti-15Mo
**Zang et al**. **(2020)**(121)	Ti-5Nb; Ti-10Nb; Ti-15Nb; Ti-20Nb; Ti-25Nb	Arc melting	cpTi: 107.4	Ti-5Nb: 77.2; Ti-10Nb: 69.2; Ti-15Nb: 87.9; Ti-20Nb: 82.1; Ti-25Nb: 77.1	Ti-10Nb< Ti-25Nb< Ti-5Nb< Ti-20Nb< Ti-15Nb< cpTi
**Zareidoost et al**. **(2021)**(200)	Ti55-Zr25-Nb10-Ta10-Ag0.7	Arc melting	NR	77 ± 4	NR
**Zhang et al**. **(2012)**(179)	Ti-7.5Nb-4Mo-xSn	Arc melting	Ti-7.5Nb-4Mo: ≈120	Ti-7.5Nb-4Mo-1Sn:≈105;Ti-7.5Nb-4Mo-2Sn:≈90; Ti-7.5Nb-4Mo-3Sn:≈90; Ti-7.5Nb-4Mo-4Sn:≈90;	Ti-7.5Nb-4Mo-2Sn; Ti-7.5Nb-4Mo-3Sn; Ti-7.5Nb-4Mo-4Sn < Ti-7.5Nb-4Mo-1Sn < Ti-7.5Nb-4Mo
**Zhang et al**. **(2015)**(189)	Ti-15Mo-5Nb; Ti-15Mo-10Nb; Ti-15Mo-15Nb	Arc melting	Ti-15Mo: 18.38	Ti-15-Mo-5Nb: 19.36, Ti-15Mo-10Nb: 18.39, Ti-15Mo-15Nb: 18.65	Ti-15Mo< Ti-15Mo-10Nb < Ti-15Mo-15Nb < Ti-15Mo-5Nb
**Zhang et al**. **(2015)**(30)	Ti-6Mo; Ti-7Mo	Arc melting	Ti-4.5Mo: 88.219	Ti-6Mo: 112.092; Ti-7Mo: 99.857	Ti-4.5Mo< Ti-7Mo< Ti-6Mo
**Zhang et al**. **(2022)**(212)	Ti-15Mo	Cold crucible levitation melting	Ti-6Al-4V: 110	Ti-15Mo:95	Ti-15Mo< Ti-6Al-4V
**Zhao et al**. **(2011)**(178)	Ti-12Mo-5Zr	Argon casting with triarc furnace	cpTi: ≈105; Ti6Al4V: ≈114	≈64.5	12Mo-5Zr < cpTi < Ti6Al4V
**Zhao et al**. **(2011)**(199)	Ti-30Zr-5Cr; Ti-30Zr-1Cr-5Mo; Ti-30Zr-2Cr-4Mo; Ti-30Zr-3Cr-3Mo	Arc melting	Ti-30Zr-4Cr: 70	Ti-30Zr-5Cr: 66; Ti-30Zr-1Cr-5Mo: 70; Ti-30Zr-2Cr-4Mo: 65; Ti-30Zr-3Cr-3Mo: 70.	Ti-30Zr-2Cr-4Mo; Ti-30Zr-5Cr; Ti-30Zr-1Cr-5Mo; Ti-30Zr-3Cr-3Mo< Ti-30Zr-4Cr
**Zhao et al**. **(2011)**(228)	Ti-30Zr-2Mo; Ti-30Zr-5Mo; Ti-30Zr-6Mo; Ti-30Zr-7Mo; Ti-30Zr-8Mo	Arc melting	Ti-30Zr: 100	Ti-30Zr-2Mo: 90; Ti-30Zr-5Mo: 80; Ti-30Zr-6Mo: 70; Ti-30Zr-7Mo: 72; Ti-30Zr-8Mo: 78	Ti-30Zr-6Mo< Ti-30Zr-7Mo< Ti-30Zr-8Mo< Ti-30Zr-5Mo< Ti-30Zr-2Mo< Ti-30Zr
**Zhao et al**. **(2012)**(45)	Ti-16Mo; Ti-17Mo; Ti-18Mo	Arc melting	Ti-15Mo: 79	Ti-16Mo: 77; Ti-17Mo: 73; Ti-18Mo: 75	Ti-17Mo< Ti-18Mo< Ti-16Mo< Ti-15Mo
**Zhao et al**. **(2020)**(188)	Ti-24Nb-4Zr-0.5Co; Ti-24Nb-4Zr-1Co; Ti-24Nb-4Zr-1.5Co	Arc melting	Ti-24Nb-4Zr: ≈52	Ti-24Nb-4Zr-0.5Co: ≈62; Ti-24Nb-4Zr-1Co: ≈68; Ti-24Nb-4Zr-1.5Co:≈75	Ti-24Nb-4Zr< Ti-24Nb-4Zr-0.5Co< Ti-24Nb-4Zr-1Co< Ti-24Nb-4Zr-1.5Co
**Mishchenko et al**. **(2020)**(40)	19Ti-59.5Zr-21.4Nb	Arc melting	NR	28.3	NR

a NR = not reported. HEBM = high-energy ball milling.

b Elastic modulus ranking represents the ranking between control and β-type Ti alloys based on the lower to the higher elastic modulus values reported.

### 4.4 Unraveling the Corrosion Behavior of β-Type Ti Alloys

Table 2 shows a comparative ranking between the control and experimental groups of β-Ti alloys in terms of corrosion resistance. The analysis includes the corrosion current density (I_corr_) associated with electrochemical corrosion processes, revealing that approximately 33% of the studies highlighted significant results in this area, showing marked differences in corrosion resistance between the different alloys investigated. A higher I_corr_ value indicates a higher corrosion rate. Comparatively, β-Ti alloys, as studied in seven publications,^5,14,86,91,136,231^ demonstrate promising corrosion resistance when compared to cpTi or Ti-6Al-4V. They are considered strong candidates to replace current α alloys and even cpTi. However, the diversity of testing solutions—such as Simulated Body Fluid (SBF),^14,42,136^ aqueous solutions,^5^ artificial saliva,^39^ hank’s solution,^86^ and Ringer solution,^91^ poses challenges in determining the optimal alloy composition and elemental proportions for specific applications.

**Table 2:** Summary of β-Type Ti Alloy Evaluated in the Included Studies Regarding the Composition, Processing Route, And Corrosion Current Density Results^a^

	**β -type Ti alloy**		**Corrosion current density (I**_**corr**_**)**		
**Author**	**Composition (wt%)**	**Processing route**	**Control**	**β alloy**	**i**_**corr**_**ranking^b^**
**Alberta et al**. **(2022)**(215)	(Ti-45Nb)96–4 Ga; (Ti-45Nb)96–4Cu; (Ti-45Nb)96–2 Ga-2Cu	Arc melting	80 ± 20 (nA cm^2^)	(Ti-45Nb)96–4Ga:88 ± 24; (Ti-45Nb)96–4Cu: 64 ± 13; (Ti-45Nb)96–2 Ga-2Cu: 69 ± 16 (nA cm^–2^)	(Ti-45Nb)96–4Cu< (Ti-45Nb)96–2 Ga-2Cu< Ti-45Nb< (Ti-45Nb)96–4 Ga
**Awwaluddin et al**. **(2020)**(217)	Zr-6Mo-4Ti-2Y; Zr-6Mo-4Ti-3Y	Arc melting	Zr-6Mo-4Ti-Y: 1.75 × 10^–7^ A	Zr-6Mo-4Ti-2Y: 1.49 × 10^–7^ ; Zr-6Mo-4Ti-3Y: 2.08 × 10^–7 A^	Zr-6Mo-4Ti-2Y< Zr-6Mo-4Ti-Y< Zr-6Mo-4Ti-3Y
**Bertrand et al**. **(2010)**(91)	Ti-25Ta-25Nb	Cold crucible semilevitation melting	cpTi: 0.822 (μA cm^–2^)	0.100 (μA cm^–2^)	Ti-25Ta-25Nb< cpTi
**Cordeiro et al**. **(2019)**(14)	Ti-35Nb-7Zr-5Ta	Arc melting	cpTi: 1.82; Ti-6Al-4V: 1.91 (nA cm^2^)	0.08 (nA cm^2^)	Ti-35Nb-7Zr-5Ta < cpTi < Ti-6Al-4V
**Fujisawa et al**. **(2018)**(180)	Ti-Nb-Sn	Arc melting	NR	NR	NR
**Gabriel et al**. **(2017)**(181)	Ti-12Mo-13Nb	Arc melting	Ti-6Al-4V: – 0.129(μA cm^–2^)	1.016(μA cm^–2^)	Ti-6Al-4V< Ti-12Mo-13Nb
**Gebert et al**. **(2015)**(229)	Ti-40Nb	Arc melting	Ti-40Nb-4In: 0.1 ± 0.2(μA cm^–2^)	0.1 ± 0.2(μA cm^–2^)	Ti-40Nb-4In= Ti-40Nb
**Guo et al**. **(2013)**(184)	Ti-35-Nb-2Ta-3Zr	Arc melting	Ti-6Al-4V: 0.52(nA cm^2^)	0.1 (nA cm^2^)	Ti-6Al-4V < Ti-35-Nb-2Ta-3Zr
**Hwang et al**. **(2021)**(187)	Ti-39Nb-6Zr-0.45Al	NR	Ti-39Nb-6Zr: 3157 (μA cm^–2^)	2104 (μA cm^–2^)	Ti-39Nb-6Zr+0.45Al< Ti-39Nb-6Zr
**Kumar et al**. **(2009)**(209)	Ti-15Mo	NR	cpTi: 32 (1 × 10^–6^); Ti-6Al-4V: 32.4 (1 × 10^–6^) (A.cm^–2^)	32.4 (1 × 10^–6^) (A.cm^–2^)	CP-Ti< Ti-6Al-4V; Ti-15Mo
**Kumar et al**. **(2011)**(87)	Ti-25-Mo	Arc melting	cpTi: 32 ± 3	10 ± 5	Ti-25-Mo< cpTi
**Kumar et al**. **(2019)**(210)	Ti-6Al-4V-2.5Cu; Ti-29Nb-13Ta-4.6Zr	NR	Ti-6Al-4V: 1.071 (μA cm^–2^)	Ti-6Al-4V-2.5Cu: 0.307; Ti-29Nb-13Ta-4.6Zr: 1.294 (μA cm^–2^)	Ti-6Al-4V-2.5Cu< Ti-6Al-4V< Ti-29Nb-13Ta-4.6Zr
**Martins Junior et al**. **(2017)**(65)	Ti-15Mo-5Nb; Ti-15Mo-10Nb; Ti-15Mo-15Nb; Ti-15Mo-20Nb	Arc melting	Ti-15Mo: 1.46 (1) × 10^–9^ (A.cm^–2^)	Ti-15Mo-5Nb: 2.57 (5) × 10^–9^; Ti-15Mo-10Nb: 4.06 (2) × 10^–9^; Ti-15Mo-15Nb: 1.08 (4) × 10^–9^; Ti-15Mo-20Nb: 6.6 (1) × 10^–9^ (A.cm^–2^)	Ti-15Mo-15Nb< Ti-15Mo< Ti-15Mo-5Nb< Ti-15Mo-10Nb< Ti-15Mo-20Nb
**Nnamchia et al**. **(2019)**(86)	Ti-8Mo-6Nb-4Zr; Ti-8Mo-5Nb-3Zr; Ti-8Mo-4Nb-2Zr; Ti-8Mo-4Nb-5Zr.	Arc melting	Ti-6Al-4V: 0.326 (μA cm^–2^)	Ti-8Mo-6Nb-4Zr: 0.214 (0.07); Ti-8Mo-5Nb-3Zr: 0.217 (0.06); Ti-8Mo-4Nb-2Zr: 0.22 (0.05); Ti-8Mo-4Nb-5Zr: 0.24 (0.05) (μA cm^–2^)	Ti-8Mo-6Nb-4Zr< Ti-8Mo-5Nb-3Zr< Ti-8Mo-4Nb-2Zr< Ti-6Al-4V
**Omran et al**. **(2020)**(204)	Ti-36Nb-3.5Sn	Arc melting	Ti-6Al-4 V: 6.9 (μA cm^–2^)	1.78 (μA cm^–2^)	Ti-36Nb-3.5Sn< Ti-6Al-4 V
**Popa et al**. **(2012)**(70)	Ti-20Nb-10Zr-5Ta	Arc melting	cpTi: 0.746 (μA cm^–2^)	0.136 (μA cm^–2^)	Ti-20Nb-10Zr-5Ta< cpTi
**Romero-Resendiz et al**. **(2023)**(21)	Ti - 15Mo - 5In	Powder metallurgy	NR	NR	NR
**Santos et al**. **(2023)**(5)	Ti-Zr; Ti-Zr-Mo	Arc melting	cp-Ti: 6.82 (μA cm^–2^)	Ti-Zr: 6.54; Ti-Zr-Mo: 6.13 (μA cm^–2^)	Ti-Zr-Mo< Ti-Zr< cp-Ti
**Saood et al**. **(2021)**(136)	Ti-7Mo-8Nb	Powder metallurgy	Ti-6Al-4V: 0.37468 (μA cm^–2^)	0.05313 (μA cm^–2^)	Ti-7Mo-8Nb < Ti-6Al-4V
**Sutowo (2020)**(223)	Ti-6Mo-6Nb-4Sn-4Mn; Ti-6Mo-6Nb-4Sn-8Mn; Ti-6Mo-6Nb-8Sn; Ti-6Mo-6Nb-8Sn-4Mn; Ti-6Mo-6Nb-8Sn-8Mn	Arc melting	Ti-6Mo-6Nb-4Sn: 3.162 (μA cm^–2^)	Ti-6Mo-6Nb-4Sn-4Mn: 0.633; Ti-6Mo-6Nb-4Sn-8Mn: 0.131; Ti-6Mo-6Nb-8Sn: 5.010; Ti-6Mo-6Nb-8Sn-4Mn: 0.112; Ti-6Mo-6Nb-8Sn-8Mn: 0.127 (μA cm^–2^)	Ti-6Mo-6Nb-8Sn-4Mn< Ti-6Mo-6Nb-8Sn-8Mn< Ti-6Mo-6Nb-4Sn-8Mn< Ti-6Mo-6Nb-4Sn-4Mn< Ti-6Mo-6Nb-4Sn< Ti-6Mo-6Nb-8Sn
**Utomo et al**. **(2023)**(177)	Ti-30Nb-2Sn; Ti-30Nb-5Sn; Ti-30Nb-8Sn.	Arc melting	Ti-30Nb: 8.25 × 10^–7^ (A.cm^–2^)	Ti-30Nb-2Sn: 3.70 × 10^–7^; Ti-30Nb-5Sn: 1.50 × 10^–7^; Ti-30Nb-8Sn: 6.59 × 10^–7^ (A.cm^–2^)	Ti-30Nb-5Sn< Ti-30Nb-2Sn< Ti-30Nb-8Sn < 8.25 X10^–7^
**Wei et al**. **(2020)**(225)	Zr-25Ti-2.5Mo; Zr-25Ti-5Mo; Zr-25Ti-7.5Mo; Zr-25Ti-10Mo; Zr-25Ti-12.5Mo	Arc melting	Zr-25Ti: 57.465 (μA cm^–2^)	Zr-25Ti-2.5Mo: 0.054; Zr-25Ti-5Mo: 0.044; Zr-25Ti-7.5Mo: 0.032; Zr-25Ti-10Mo: 0.038; Zr-25Ti-12.5Mo: 0.039 (μA cm^–2^)	Zr-25Ti-7.5Mo< Zr-25Ti-10Mo< Zr-25Ti-12.5Mo< Zr-25Ti-5Mo< Zr-25Ti-2.5Mo< Zr-25Ti
**Wu et al**. **(2015)**(226)	Ti60-Zr10-Si15-Ta12-Nb3; Ti60-Zr10-Si15-Ta8-Nb7; Ti60-Zr10-Si15-Ta4-Nb11	Arc melting	Ti60-Zr10-Si15-Ta15: 0.291 ± 0.022 (μA cm^–2^)	Ti60-Zr10-Si15-Ta12-Nb3: 0.554 ± 0.052; Ti60-Zr10-Si15-Ta8-Nb7: 0.297 ± 0.004; Ti60-Zr10-Si15-Ta4-Nb11: 0.301 ± 0.002 (μA cm^–2^)	Ti60-Zr10-Si15-Ta15< Ti60-Zr10-Si15-Ta8-Nb7< Ti60-Zr10-Si15-Ta4-Nb11< Ti60-Zr10-Si15-Ta12-Nb3
**Wu et al**. **(2022)**(3)	Ti-24Nb-4Zr-1Mn; Ti-24Nb-4Zr-3Mn; Ti-24Nb-4Zr-5Mn	Arc melting	Ti-24Nb-4Zr: 15.02 ± 0.48	Ti-24Nb-4Zr-1Mn: 15.38 ± 0.31; Ti-24Nb-4Zr-3Mn:27.28 ± 0.34; Ti-24Nb-4Zr-5Mn: 14.61 ± 0.28	Ti-24Nb-4Zr-5Mn< Ti-24Nb-4Zr< Ti-24Nb-4Zr-1Mn< Ti-24Nb-4Zr-3Mn
**Xie et al**. **(2015)**(90)	Ti-6Mo; Ti-8Mo; Ti-10Mo	Arc melting	Ti-4Mo: 1.25 (μA cm^–2^)	Ti-6Mo: 1.38; Ti-8Mo: 2.74; Ti-10Mo: 9.8. (μA cm^–2^)	Ti-4Mo< Ti-6Mo< Ti-8Mo< Ti-10Mo
**Xu et al**. **(2020)**(42)	Ti-35Zr-28Nb	Arc melting	cpTi: 63.31 ± 1.21; Ti-6Al-4V: 95.31 ± 1.56 (nA cm^2^)	0.057 ± 0.001 (nA cm^2^)	Ti35-Zr-28Nb< cpTi< Ti6Al4V
**Xu et al**. **(2022)**(20)	Ti-10Mo-28Nb-1Zr; Ti-10Mo-28Nb-3Zr; Ti-10Mo-28Nb-5Zr; Ti-10Mo-28Nb-7Zr	Arc melting	Ti-10Mo-28Nb: 3.901 (A.cm^–2^)	Ti-10Mo-28Nb-1Zr: 7.032; Ti-10Mo-28Nb-3Zr: 2.962; Ti-10Mo-28Nb-5Zr: 2.685; Ti-10Mo-28Nb-7Zr: 2.790. (A.cm^–2^)	Ti-10Mo-28Nb-5Zr< Ti-10Mo-28Nb-7Zr< Ti-10Mo-28Nb-3Zr< Ti-10Mo-28Nb< Ti-10Mo-28Nb-1Zr: 7.032
**Zhang et al**. **(2020)**(231)	Ti-Mo-Ag	Arc melting	cpTi:0.0 52 ± 0.006 (nA cm^2^)	0.023 ± 0.0028 (nA cm^2^)	Ti-Mo-Ag< cpTi
**Zhao et al**. **(2011)**(178)	Ti-12Mo-5Zr	Argon casting with triarc furnace	cpTi: – 0.62; Ti6Al4V: – 6.05 (μA cm^–2^)	–3.70 (μA cm^–2^)	Ti6Al4V< Ti-12Mo-5Zr < cpTi
**Zhao et al**. **(2020)**(188)	Ti-24Nb-4Zr-0.5Co; Ti-24Nb-4Zr-1Co; Ti-24Nb-4Zr-1.5Co	Arc melting	Ti-24Nb-4Zr: 0.44 (A.cm^–2^)	Ti-24Nb-4Zr-0.5Co: 0.93; Ti-24Nb-4Zr-1Co: 0.98; Ti-24Nb-4Zr-1.5Co:0.65 (A.cm^–2^)	Ti-24Nb-4Zr< Ti-24Nb-4Zr-1.5Co < Ti-24Nb-4Zr-0.5Co < Ti-24Nb-4Zr-1Co
**Zhou et al**. **(2005)**(198)	Ti-10Ta; Ti-30Ta; Ti-70Ta	Arc melting	cpTi: – 0,6; Ti-6Al-4V ELI: – 0,5 (μA cm^–2^)	Ti-10Ta: – 0,6; Ti-30Ta: 1,8; Ti-70Ta: 1,8(μA cm^–2^)	cpTi< Ti-6Al-4V ELI< Ti-10Ta< Ti-30Ta; Ti-70Ta

a NR = not reported. HEBM = high-energy ball milling.

b Corrosion current density ranking represents the ranking between control and β-type Ti alloys based on the lower to the higher corrosion current values reported.

In the reviewed studies, alloy composition diversity significantly influences corrosion resistance. Utomo et al., 2023^177^ demonstrated that adding Sn to Ti-Nb alloys, particularly in Ti-Nb-Sn ternary alloys, enhances corrosion resistance, particularly effective with arc melting. Conversely, Zhao et al., 2020^188^ found that adding Co to Ti-24Nb-4Zr-xCo alloys increases corrosion susceptibility due to ω phase precipitation and film destabilization. In contrast, Xu et al., 2022^20^ showed that increasing Zr content in Ti-Mo-Nb-xZr alloys results in slower corrosion rates, forming a stable, protective ZrO_2_ layer at grain boundaries. This natural oxide layer enhances corrosion resistance as Zr content increases on the alloy surface.^5^

Wu et al., 2015^226^ found that a quaternary alloy (Ti-60Zr-10Si-15Ta) exhibited improved corrosion resistance in the absence of Nb due to its homogeneous microstructure without crystalline defects or second-phase precipitates, resulting in stable and protective passive films.^233^ Wei et al., 2020^225^ demonstrated that adding 7.5 wt% Mo to Zr-25Ti alloys reduced current density by about 44% by forming a protective oxide layer through passivation. Additionally, Wu et al., 2022^3^ highlighted that increasing Mn content in alloys improved corrosion resistance, with a 5% addition showing enhanced anticorrosive properties and a larger capacitive radius.

### 4.5 Exploring Alloying Elements: Impact on Elongation and Hardness

An analysis of elongation and hardness in Ti alloys (Table S2) provides key insights into their mechanical properties crucial for orthopedic implants and medical devices. Comparing alloys like cpTi and Ti-6Al-4V reveals that cpTi exhibits greater ductility, indicating higher elasticity before fracture, while sensitivity to heat treatments varies.^178^ Nnamchia et al., 2016^86^ found that β-type Ti alloys, like Ti-8Mo-4Nb-5Zr, show a 10% higher elongation (26%) compared to Ti-6Al-4V (16%). Hacisalihoglu et al., 2015^186^ report intermediate elongation values for β-type Ti alloys: Ti-13Nb-13Zr (28%) and Ti-15Mo (17%), balancing with desired properties such as hardness (265 HV and 360 HV, respectively). Furthermore, adding more β stabilizers, like Mo in Ti-30Zr-Cr-Mo, enhances ductility, suggesting effective optimization of mechanical properties for implant applications.^186^

It should also be noted that elongation values reflect the ability of materials to undergo plastic deformation, with alloys such as Ti-30Zr-Mo showing high ductility, reaching 40% when using a higher concentration of Mo (Ti-30Zr-1Cr-5Mo).^178^ Thus, we observed that in addition to the microstructure, the chemical composition directly influences these results, as seen in Ti-22Zr-11Nb-2Sn, with greater elongation (14.5%) compared to the non- β control (3.8%) due to its favorable composition and microstructure.^22^

In addition, authors^70,177,182,214,217^ have shown that the hardness of titanium alloys is subject to a multitude of factors that interact in a complex way and dictate the mechanical properties of these materials, the main one being the chemical composition depending on the different additions of β elements in the alloy, such as Nb, Mo, Sn and Co.^70,177,182,214,217^ In their study, Kumar et al., 2009^209^ compared the hardness of cpTi and the β Ti-15Mo alloy, finding 175 HV and 338 HV respectively, demonstrating that the β-type Ti alloy does indeed have greater hardness than commonly used materials due to the introduction of these elements. Popa et al., 2012^70^ found a hardness of 145 HV for cpTi and 236 HV for the β alloy Ti-20Nb-10Zr-5Ta and Almeida et al., 2020^216^ found a value of approximately 77 HV for cpTi and 207 HV for Ti-12Mo-25Nb. The β Ti-16Nb alloy has a hardness of 338 HV, a higher value than cpTi, which is further enhanced after the inclusion of the β stabilizer Zr, becoming the Ti-16Nb-15Zr alloy and increasing to 415 HV.^227^ The same behavior was observed found a hardness of 398 ± 6 HV for the Ti-24Nb-4Zr alloy, a value which increased to 437 ± 9 HV after adding more β stabilizer elements Ti-24Nb-4Zr-3Mn, reinforcing the positive influence of these elements on hardness.^3^ However, to achieve these positive results, the microstructure of the powder mixture must be homogeneous.^234,235^ This can be challenging due to differences in particle size, melting point, and density of the metallic elements, which can lead to powder segregation, partial melting, and instability in the melt pool.^234,235^ Therefore, parameters such as scanning speed, laser power, powder layer thickness, and scanning space are crucial to the quality of the final product.^236^

In addition to the chemical composition, heat treatments and manufacturing processes play a fundamental role in determining the hardness of titanium alloys. The impact of varying heat treatment temperatures was explored, revealing that elevating the temperature correspondingly enhances the substrate’s hardness, with the control Ti-6Al-4V showing ≈341 HV and the experimental Ti-12Mo-5Zr (1053K ST) ≈427 HV.^178^ However, excessively high temperatures can reduce this value, finding a lower hardness of ≈442 HV when increasing the temperature to 1133K, thus highlighting the criticality of meticulous control of processing conditions to achieve the desired mechanical properties.^178^

### 4.6 High Entropy or Multiprincipal Alloys for Biomedical Applications

The evaluation of entropy plays a fundamental role in the characterization and development of advanced materials, especially high entropy alloys (HEAs) and multiprincipal alloys. The entropy of mixing (ΔSm) defines the structural complexity of these materials and directly influences their mechanical, thermal, and even biological properties. Understanding and controlling entropy is essential for optimizing the structure and properties of these alloys, paving the way for innovative applications in various areas. For example, in biomedical engineering, where there is a growing demand for materials with specific characteristics, such as lower modulus of elasticity and greater biocompatibility, the manipulation of entropy could offer promising solutions.^227,237,238^

From the point of view of mixing entropy (ΔSm), materials can be classified into 3 classes: low entropy (1 or 2 components); medium entropy (3 or 4 components), and high entropy (5 or more components).^84^ Briefly, HEAs are composed of at least 5 main elements, with an atomic percentage ranging from 5 to 35%. The addition of multiple main components increases the entropy of the system ΔSm, to form a single-phase alloy in a solid solution (e.g., BCC in the case of β Ti alloys or composed by β-stabilizing elements: Ti, Nb, Mo, Ta, and others), which reduces the number of phases predicted by the Gibbs rule, making the structure simpler and expected to present superior and optimized properties (effect of high entropy).^227^

Unlike conventional alloys where atoms primarily bond with atoms of the same species, HEAs feature asymmetric chemical bonds due to atoms bonding with different atomic sizes and crystalline structures. This asymmetry distorts the material’s crystalline structure, influencing its mechanical properties.^227^ Moreover, HEAs exhibit slower diffusion rates, which delay nucleation and growth of new phases. This slower diffusion, however, facilitates the refinement of precipitates, enhances control over grain size, and elevates the material’s recrystallization temperature.^227,237^ Although ΔSm is maximum when the elements are in equiatomic proportion, alloys with this composition generally have a high elastic modulus and, in the case of the Ti-Nb-Zr-Ta-Mo system, they have a high melting point (refractory alloys) since these alloys are based on Ta and Mo due to the high atomic weight of these elements. In this sense, it is necessary to develop HEAs alloys with a lower elastic modulus for biomedical application (BioHEA), and, therefore, equimassic BioHEAs can be an alternative.^238^

Research on new BioHEAs primarily focuses on microstructure, corrosion resistance, tensile strength, and ductility improvement, with limited attention to elastic modulus due to its typically higher values compared to cpTi and Ti-6Al-4V alloys. Promising results indicate properties superior to traditional alloys, yet further development is needed. Equiatomic alloys in the Ti-Nb-Zr-Ta-Mo system tend to exhibit refractory behavior with high elastic modulus (E = 116 - 153 GPa). Researchers are exploring medium entropy alloys or equimolar multiprincipal BCC alloys with multiple β-stabilizing elements within an optimal entropy range (1.1 < ΔSm < 1.3R), such as Ti-33Nb-33Zr, Ti-25Nb-25Zr-25Ta, and Ti-20Nb-20Zr-20Ta-20Mo (wt.%), offering elastic modulus values of E = 73 - 88 GPa alongside enhanced mechanical strength.^5^

### 4.7 Cytocompatibility and Microbiological Performance of β-Type Ti Alloys

It is understood that evaluating the mechanical properties of β-type Ti alloys is crucial since the elastic modulus plays a fundamental role in the implantation process in vivo, highlighting the importance of reducing it for the development of suitable dental implants. However, it is equally important not to neglect the evaluation of the biological and microbiological properties of these alloys to ensure the effectiveness of their clinical applicability. Although the main results of the systematic search were elastic modulus and hardness, this review identified a limited number of studies that also addressed biological aspects, representing approximately 24% of the total number of articles analyzed. Of these, around 19.5% focused on biological analyses, only 2% addressed microbiological aspects and 2% investigated both analyses.

Biological analyses consistently show β-type Ti alloys promoting favorable outcomes in cell adhesion and bone formation. Fujisawa et al., 2018^180^ observed bone formation in both groups studied, highlighting the β Ti-25.4Nb-9.9Sn alloy’s ability to prevent cortical bone atrophy. Hwang et al., 2021^187^ found thinner fibrous tissue correlated with enhanced biocompatibility, with TNZA and TNZ40 alloys showing superior results with membrane thicknesses of 82.3 and 62.5 μm, respectively. Niinomi et al., 1999^206^ noted greater direct bone contact with the Ti-29Nb-13Ta-4.6Zr alloy compared to Ti-6Al-4V and SUS 316 L stainless steel.

Guo et al., 2013^184^ emphasize that the topographical properties and elemental composition significantly impact cell behavior. They found that Ti-35Nb-2Ta-3Zr, with a higher average roughness (Ra) than Ti-6Al-4V, exhibited greater biocompatibility, promoting enhanced adhesion, spreading, proliferation, and differentiation of MC3T3-E1 cells. Furthermore, they observed increased regulation and expression of genes associated with TGFβ and BMP signaling pathways (BMP2, BMP6, SP1, CREBBP, RBL2, TBS3, ACVR1, and ZFYVE16) (132). Similarly, Kopova et al., 2016^69^ demonstrated that cells cultured on TNZT alloys with β stabilizers such as 2Fe, 0.5Si + 1Fe, and 0.5Si + 2Fe (by weight) exhibited higher collagen I production compared to polystyrene culture plates. They also noted superior differentiation of HOB-p cells based on collagen I production compared to Ti-6Al-4V alloy.^69^

**Figure 10:**
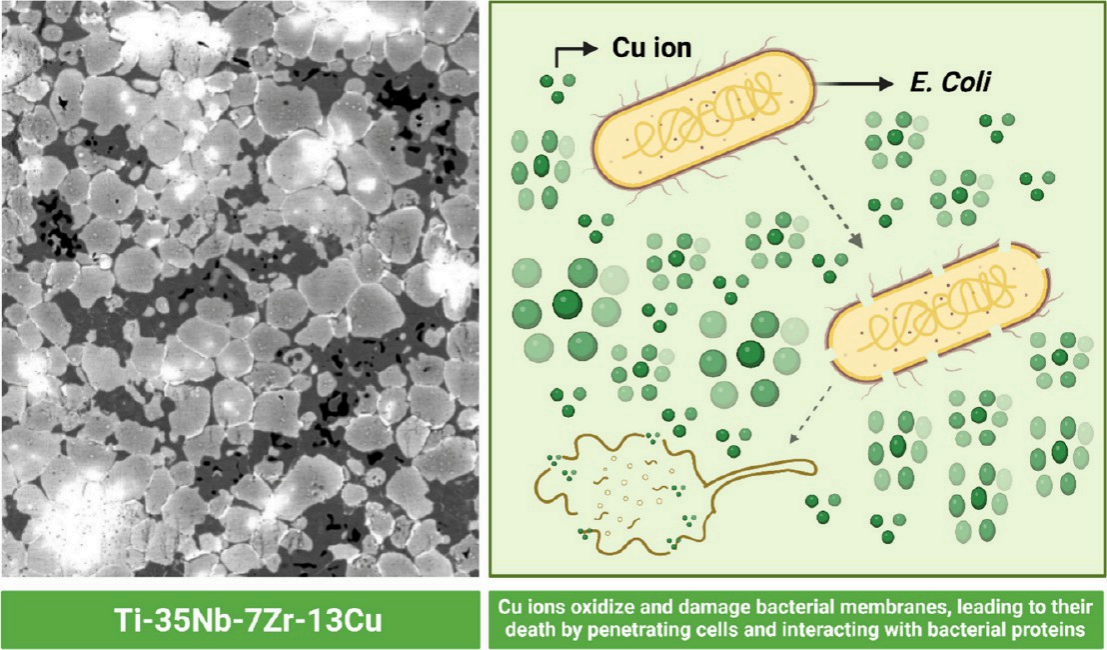
Illustration of the mechanism of action of Cu ions against bacteria, involving the rupture of cell membranes and reaction with bacterial proteins. Reproduced or adapted with permission from [^163^]. Copyright [2016] [Elsevier]. Created with BioRender.com (License number: WP272L85K5).

Regarding microbiological tests, only three studies addressed related results, all of which were considered positive. Yi et al., 2021^163^ and Kumar et al., 2009^209^ investigated β-type Ti alloys with added copper, finding a significant improvement in bacterial resistance. In addition, Yi et al., 2021^163^ observed a reduction in the formation of colonies of bacteria, such as *E*. *coli* and *S*. *aureus*, on the surface of the alloy in the presence of copper, with antibacterial rates of over 99.99% for copper contents of 10% and 13%, indicating a direct relationship between copper content and antibacterial efficacy, which possibly causes oxidative damage to bacteria. As described by this author,^163^ Cu ions are released and come into contact with the bacteria, breaking their cell membranes. These ions manage to penetrate the bacterial cells and react with their proteins, resulting in their death, as illustrated in figure 10.

## 5 Facing Forward: Current Challenges and Future Perspectives

Based on the insights gleaned from the reviewed articles, β alloys represent a promising avenue for advancing the development of more effective and safer biomedical implants. These alloys exhibit a lower elastic modulus than currently available implants, thereby mitigating tension and bone stress while demonstrating corrosion resistance. Moreover, depending on their surface composition, they may promote antimicrobial activity and enhance biological safety in various applications. However, significant challenges persist, necessitating further exploration in several key areas:

1) Efficient processing and postprocessing routes to achieve the desired mechanical properties.
2) Composition and elemental ratios that optimize physical, chemical, electrochemical, mechanical, biological, and microbiological properties for large-scale production.
3) Antimicrobial effects and evaluation methodology: Additional detailed analysis using advanced methodologies, particularly on biofilm models that replicate the oral environment, is imperative to ensure clinically relevant results regarding efficacy and safety.
4) Toxicity: Further investigation into genotoxicity and cytotoxicity, employing reliable models and various human cell lines, is essential for comprehensively understanding the risks associated with these materials.
5) Substrate and osseointegration: While β alloys hold significant promise for the advancement of biomedical and dental implants, additional in vivo research correlating bone condition, implant macro geometry, and osseointegration is necessary to address identified challenges and fill gaps in scientific knowledge.

## 6 Conclusion

β-Ti alloys represent a promising innovation in the field of dental implants, to overcome the limitations presented by α and α+β alloys. Below are the main points highlighted in this review:

- Optimization of properties: Variations in processing methods, postprocessing techniques, and element composition optimize the alloys’ physical and mechanical properties, resulting in a modulus of elasticity closer to that of bone and greater corrosion resistance.
- Compositional adjustments: The controlled addition of β elements, such as Nb, Mo, Sn, Hf, or Ta, favors the customization of the mechanical and electrochemical characteristics of the alloys, adapting them to the oral environment.
- Biological performance: Studies reviewed indicate that some β-Ti alloys promote cell adhesion and have antibacterial properties. However, the evidence on antibacterial activity is not consistent, and few studies have delved into these aspects. It is important to mention that alloys without antimicrobial elements are not designed to demonstrate antibacterial activity.
- Innovation potential: The ability to adjust the composition of β-Ti alloys to meet the specific needs of the oral environment reveals their great potential to transform the practice of dental implants. This adjustment allows for an optimal balance of mechanical strength, elasticity, and corrosion resistance, improving implant durability and promoting more effective biological integration, as well as a more favorable immune response.
- Need for further research: Despite the advances, the diversity in manufacturing processes, postprocessing, and methodologies for evaluating electrochemical properties and modulus of elasticity still prevents a definitive conclusion on the ideal combination of elements, composition, and production route. Therefore, further studies are needed to exploit the clinical potential of β-Ti alloys in dental implantology.

## Supporting Information Available

Materials and methods; tables showing the search strategy and summary of the β-type Ti alloys evaluated (PDF)
