# Supplementary material for: Recent Advances and Prospects in β-type Titanium Alloys for Dental Implants Applications
Source: ACS Biomater Sci Eng. 2024 Aug 31;10(10):6029–60. doi: 10.1021/acsbiomaterials.4c00963 (PMC11480944; doi:10.1021/acsbiomaterials.4c00963)
Supplement: Supplementary file 1 — ab4c00963_si_001.pdf [file ab4c00963_si_001.pdf]

Supplementary Information

Recent Advances and Prospects in β-Type Titanium Alloys for Dental Implants Applications

João V. C. Neto[1], Cícero A. S. Celles[1], Catia S. A. F. de Andrade[1], Conrado R. M. Afonso[2], Bruna E. Nagay[1] & Valentim A. R. Barão[1,*]

[1] Department of Prosthodontics and Periodontology, Piracicaba Dental School, University of Campinas (UNICAMP), Piracicaba, São Paulo 13414-903, Brazil

[2] Department of Materials Engineering (DEMa), Universidade Federal de São Carlos (UFSCar), São Carlos, São Paulo 13565-905, Brazil

*Corresponding author

Valentim Adelino Ricardo Barão

University of Campinas (UNICAMP), Piracicaba Dental School, Department of Prosthodontics and Periodontology, Av. Limeira, 901, Piracicaba, São Paulo, 13414-903, Brazil.

vbarao@unicamp.br

1. SUPPLEMENTARY MATERIAL AND METHODS

The review process began with an electronic search conducted in February 2024 by three independent examiners (J.V.C.N., C.A.S.C., and C.S.A.F.A.) across four electronic databases: PubMed (MEDLINE), Scopus, Web of Science, and EMBASE. MeSH terms, entry terms, and free terms were employed, with adjustments made to adhere to the syntax rules of each respective database (Table S1). Search alerts were established to ensure the ongoing relevance of the search strategy. Additionally, a manual screening of the bibliographies of all included studies was performed to supplement the electronic search. Notably, no language or publication date restrictions were applied during the search process, ensuring inclusivity of relevant literature across various languages and publication years.

Inclusion criteria for this review encompassed studies conducted in vitro, pre-clinical, or clinical settings. Specifically, the focus was on beta-type titanium alloys and the substrate under investigation needed to target biomedical or dental implants, with clarity in the abstract or title regarding this application. The primary outcomes of interest included the elastic modulus (Young modulus) and corrosion resistance, both of which should be clearly reported or evaluated. Exclusion criteria were applied to reviews, conference abstracts, technical reports, letters to editors, opinions, theses, book chapters, symposium reports, association reports, and unpublished studies. Additionally, studies examining titanium alloys other than beta-type, and those not evaluating elastic modulus or corrosion resistance, were excluded from consideration.

In the initial stage of the review process, all references obtained from the databases were imported into the Rayyan QCRI reference manager. Following the removal of duplicates, three authors (J.V.C.N., C.A.S.C., and C.S.A.F.A.) independently screened all titles and abstracts according to the predetermined inclusion criteria. If the information provided in the titles and abstracts was insufficient to determine eligibility, the full texts of the articles were retrieved and reviewed to prevent the inadvertent exclusion of relevant studies. Subsequently, the full texts of the selected studies underwent independent assessment by the reviewers. Any discrepancies or disagreements between the reviewers were resolved through open discussion. If consensus could not be reached, a third author (B.E.N.) was consulted to facilitate resolution and ensure consistency in the selection process.

Data extracted from the included studies covered various aspects, including alloy composition, processing routes, control groups, elastic modulus, corrosion resistance, compressive strength, elongation, and hardness. Additionally, when studies assessed the microbiological and biological properties, they were documented as well. Each study's findings were assessed independently to ascertain their individual

contributions. Mechanical and electrochemical outcomes were characterized by mean/median and standard deviation values, whenever available. In cases where exact values were not provided but data were presented graphically, the WebPlotDigitizer program was employed for accurate data extraction. Furthermore, secondary outcomes related to microbiological and biological data were qualitatively evaluated. The results were systematically organized using tables.

2. SUPPLEMENTARY TABLES

Table S1. Search strategy (February 02, 2024).

MEDLINE - PubMed (n=494)	((alloy[MeSH Terms] OR alloy, dental[MeSH Terms] OR titanium alloy[Title/Abstract] OR alloy*[Title/Abstract]) AND (beta-type[Title/Abstract] OR beta[Title/Abstract] OR β[Title/Abstract])) AND (implant*[Title/Abstract])
Scopus (n=1,975)	(TITLE-ABS-KEY (titanium AND alloy) OR TITLE-ABS-KEY (alloy*)) AND (TITLE-ABS-KEY (beta-type) OR TITLE-ABS-KEY (beta) OR TITLE-ABS-KEY (β)) AND (TITLE-ABS-KEY (implant*))
Web of Science (n=1,791)	(TS=(titanium alloy)) OR TS=(alloy*)<br>AND<br>((TS=(beta-type)) OR TS=(beta)) OR TS=(β)<br>AND<br>TS=(implant*)
Embase (n=422)	'titanium alloy':ti,ab,kw OR 'alloy*':ti,ab,kw<br>AND<br>'beta-type':ti,ab,kw OR 'beta':ti,ab,kw OR 'β':ti,ab,kw<br>AND<br>'implant*':ti,ab,kw

Legend: TS: topic; Ti: Title; Ab: Abstract; kw: Keywords

Table S2. Summary of beta-type Ti alloys evaluated in the included studies regarding the composition, processing route, elongation and hardness results.

Author	Beta-type Ti alloy		Elongation (%)			Hardness (VHN) cordeiro		
	**Composition (wt%)**	**Processing route**	**Control**	**Beta alloy**	**Elongation ranking**	**Control**	**Beta alloy**	**Hardness ranking**
Cordeiro et al., 2019[14]	Ti-35Nb-7Zr-5Ta	Arc melting	NR	NR	NR	cpTi Machined: ≈187 TiAlV PEO: graph	Machined: ≈194 PEO: graph	Ti-35Nb-7Zr-5Ta >cpTi
Utomo et al.,2023[177]	Ti-30Nb-2Sn; Ti-30Nb-5Sn; Ti-30Nb-8Sn.	Arc melting	NR	NR	NR	NR	Ti-30Nb: 362.9; Ti-30Nb-2Sn: 392.1; Ti-30Nb-5Sn: 312.2; Ti-30Nb-8Sn: 365.3	Ti-30Nb-2Sn > Ti-30Nb-8Sn > Ti-30Nb > Ti-30Nb-5Sn
Zhao et al.,2011[178]	Ti-12Mo-5Zr	Arc melting	cpTi: 20; Ti6Al4V:15	Ti-12mo-5zr (H): ≈4.1; Ti-12mo-5zr (1053K ST): ≈3.9; Ti-12mo-5zr (1133K ST):≈4.7	Ti-12Mo-5Zr (1133K ST) > Ti-12Mo-5Zr (H) > Ti-12Mo-5Zr (1053K ST) > CpTi > Ti6Al4V.	cpTi: 145; Ti6Al4V:34 1	Ti-12Mo-5zr (H): ≈396; Ti-12Mo-5zr (1053K ST): ≈427; Ti-12Zo-5zr (1133K ST): ≈442	Ti-12Mo-5Zr (1133K ST) > Ti-12Mo-5Zr (1053K ST) > Ti-12Mo-5Zr (H) > Ti6Al4V > CpTi
Zhang et al.,2012[179]	Ti-7.5Nb-4Mo-xSn	Arc melting	NR	NR	NR	NR	Ti-7.5Nb-4Mo: ≈290; Ti-7.5Nb-4Mo-1Sn:≈280; Ti-7.5Nb-4Mo-2Sn:≈270; Ti-7.5Nb-4Mo-3Sn:≈300; Ti-7.5Nb-4Mo-4Sn:≈300;	Ti-7.5Nb-4Mo-3Sn > Ti-7.5Nb-4Mo-4Sn > Ti-7.5Nb-4Mo > Ti-7.5Nb-4Mo-1Sn > Ti-7.5Nb-4Mo-2Sn
Golasinski et al., 2021[182]	Ti-36Nb-2Ta-3Zr-0.3O	Powder metallurgy	NR	NR	NR	Ti6Al4V: ≈6.71	Ti-36Nb-2Ta-3Zr-0.3O 1/2: ≈5.06; Ti-36Nb-2Ta-3Zr-0.3O 3: ≈4.77;	Ti6Al4V> Ti-36Nb-2Ta-3Zr0.3O ½> Ti36Nb2Ta3Zr0.3O 3:
Hacisalihoglu et al., 2015[186]	Ti-13Nb-13Zr; Ti15; Ti45	Electrode discharge machine (wire-EDM).	cp-Ti: ≈36; Ti6Al4V: ≈18	Ti1313: ≈28, Ti15:≈17, Ti45:≈21	cpTi> Ti1313> Ti45> Ti6Al4V> Ti15	cp-Ti: ≈220; Ti6Al4V: ≈400	Ti-13Nb-13Zr: ≈265, Ti15:≈360, Ti45:≈185	Ti6Al4V> Ti15> Ti1313> cp-Ti> Ti45
Hwang et al.,2021[187]	Ti–39Nb-6Zr+0.45Al	NR	Ti-39Nb-6Zr: 21.3	Ti–39Nb–6Zr+0.45Al: 20.9	Ti-39Nb-6Zr> Ti–39Nb–6Zr+0.45Al	NR	NR	NR

Zhao et al., 2020[188]	Ti-24Nb-4Zr-0.5Co; Ti-24Nb-4Zr-1Co; Ti-24Nb-4Zr-1.5Co	Arc-melting	Ti-24Nb-4Zr: ≈23	Ti-24Nb-4Zr-0.5Co: ≈22; Ti-24Nb-4Zr-1Co: ≈21; Ti-24Nb-4Zr-1.5Co:≈19	Ti-24Nb-4Zr> Ti-24Nb-4Zr-0.5Co> Ti-24Nb-4Zr-1Co> Ti-24Nb-4Zr-1.5Co	Ti-24Nb-4Zr-0.5Co: ≈22; Ti-24Nb-4Zr-1Co: ≈21; Ti-24Nb-4Zr-1.5Co:≈19	Ti-24Nb-4Zr-0.5Co: ≈190; Ti-24Nb-4Zr-1Co: ≈216; Ti-24Nb-4Zr-1.5Co:≈222	Ti-24Nb-4Zr-1.5Co > Ti-24Nb-4Zr-1Co > Ti-24Nb-4Zr-0.5Co.
Zhang et al., 2015[30]	Ti-15Mo-5Nb; Ti-15Mo-10Nb; Ti-15Mo-15Nb	Arc melting	NR	NR	NR	Ti15Mo: 334,	Ti15Mo5Nb: 323, Ti15Mo10Nb: 272, Ti15Mo15Nb: 262	Ti15Mo> Ti15Mo5Nb> Ti15Mo10Nb> Ti15Mo15Nb
Romero-Resendiz et al., 2023[21]	Ti – 15Mo – 5In	Powder metallurgy	NR	NR	NR	NR	Ti – 15Mo – 5In: ≈3.9	NR
Santos et al., 2023[192]	Ti–Zr; Ti–Zr–Mo	Arc melting	NR	NR	NR	cp-Ti: 201.21	Ti–Zr: 241.84; Ti–Zr–Mo: 292.24	Ti–Zr–Mo:> Ti–Zr> cp-Ti
Santos et al., 2023[232]	Ti-27Nb-39Zr Ti-30Nb-50Zr Ti-20Nb-30Zr-13Ta	Arc melting	NR	NR	NR	Ti-Nb: AC: 226 RH:181	50: AC:245, HR: 237; 39: AC:228, HR:227; 30: AC:226, HR:181	50: AC > HR, 39: AC = HR > 30: AC > HR.
Schaal et al., 2023[22]	Ti-22Zr-11 Nb-2Sn	Laser Powder Bed Fusion	Ti-6Al-4V ELI: 3.8%	Ti-22Zr-11 Nb-2Sn:14.5%	Ti-22Zr-11 Nb-2Sn> Ti-6Al-4V	NR	NR	NR
Cui et al., 2009[195]	Ti–28Nb–13Zr–0.5Fe	Vacuum electromagnetism induction furnace	NR	13%		NR	NR	NR
Dai et al., 2016[196]	Ti-24Nb-4Zr-8Sn	NR	NR	NR		Ti: ≈4.6; Ti-MAO: ≈2.2	Ti–24Nb–4Zr– 8Sn: ≈4.0; Ti–24Nb–4Zr– 8Sn -MAO: 0.2	Ti> Ti–24Nb–4Zr–8Sn >Ti-MAO> Ti–24Nb–4Zr– 8Sn -MAO

Dang et al., 2020[197]	Ti-2Zr-0.1Nb-0.1Sn; Ti-2Zr-0.2Nb-0.2Sn; Ti-2Zr-0.3Nb-0.3Sn; Ti-2Zr-0.1Nb-0.1Mo; Ti-2Zr-0.2Nb-0.2Mo; Ti-2Zr-0.3Nb-0.3Mo.	Arc melting	Ti-2Zr: 15.15	Ti-2Zr-0.1 Nb-0.1Sn: 27.10; Ti-2Zr-0.2Nb-0.2Sn: 47.12; Ti-2Zr-0.3Nb-0.3Sn: 49.31; Ti-2Zr-0.1Nb-0.1Mo: 46.45; Ti-2Zr-0.2Nb-0.2Mo: 46.94; Ti-2Zr-0.3Nb-0.3Mo: 46.56	Ti-2Zr-0.3Nb-0.3Sn > Ti-2Zr-0.2Nb-0.2Sn > Ti-2Zr-0.3Nb-0.3Mo > Ti-2Zr-0.2Nb-0.2Mo > Ti-2Zr-0.1Nb-0.1Mo > Ti-2Zr-0.1Nb-0.1Sn > Ti-2Zr	NR	NR	NR
Zhou et al., 2005[198]	Ti–10Ta; Ti–30Ta; Ti–70Ta	Arc melting	NR	NR		Ti–6Al–4V ELI: 250 HV	Ti–10%Ta: 160 HV; Ti–30%Ta: 170 HV; Ti–70%Ta: 200 HV	Ti–70%Ta > Ti–6Al–4V ELI > Ti–30%Ta > Ti–10%Ta.
Zhao et al., 2011[199]	Ti–30Zr–5Cr; Ti–30Zr–1Cr–5Mo; Ti–30Zr–2Cr–4Mo; Ti–30Zr–3Cr–3Mo.	Arc melting	10%	Ti–30Zr–5Cr: 5%; Ti–30Zr–1Cr–5Mo: 40%; Ti–30Zr–2Cr–4Mo: 33% ; Ti–30Zr–3Cr–3Mo: 18%		NR	NR	NR
Zareidoost et al., 2021[200]	TTi-55Zr-25Nb-10Ta-10Ag0.7	Arc melting	NR	NR	NR	NR	3,2 ± 2 Gpa	NR
Yılmaz et al., 2018[227]	Ti-16-Nb-15Zr	Arc melting	NR	NR	NR	Ti16Nb: 338 HV	Ti16Nb15Zr : 415 HV	Ti16Nb15Zr > Ti16Nb.
Yilmaz et al., 2017[202]	Ti-16Nb-2Sn; Ti-16Nb-4Sn	Arc melting	NR	NR	NR	Ti-16Nb: 6250 MPa	Ti-16Nb-2Sn: 4500 MPa; Ti-16Nb-4Sn: 5000 Mpa	Ti-16Nb > Ti-16Nb-4Sn > Ti-16Nb-2Sn.
Popa et al., 2012[70]	Ti-20Nb-10Zr-5Ta	Arc melting	NR	NR	NR	cpTi: 145 HV	Ti-20nb-10zr-5ta : 236 HV	Ti-20Nb-10Zr-5Ta > cpTi.
Okulov et al., 2013[205]	Ti-71.8Nb-14.1Al-6.7Cu-4Ni3.4	Arc melting	Ti68.8Nb13.6Al6.5Cu6Ni5.1: 3.7 % ± 0.2	Ti71.8nb14.1al6.7cu4ni3.4: 4.6 % ± 0.2	Ti71.8nb14.1al6.7cu4ni3.4 > Ti68.8Nb13.6Al6.5Cu6Ni5.1.	NR	NR	NR
Nunes et al., 2023[89]	Ti-29Nb-2Mo-3Zr;	Arc melting	NR	NR		Ti-6Al-4V: 338 HV ± 6.0	Ti-29Nb-2Mo-3Zr: 212.2 HV ± 7.0; Ti-29Nb-2Mo-6Zr: 228.4	Ti-6Al-4V > Ti-24Nb-4Mo-6Zr > Ti-24Nb-4Mo-3Zr > Ti-29Nb-

	Ti-29Nb-2Mo-6Zr; Ti-24Nb-4Mo-3Zr; Ti-24Nb-4Mo-6Zr						HV ± 5.6; Ti-24Nb-4Mo-3Zr: 235.2 HV ± 4.8; Ti-24Nb-4Mo-6Zr: 244.8 ± 9.0 HV	2Mo-6Zr > Ti-29Nb-2Mo-3Zr.
Nnamchi et al., 2016[86]	Ti–8Mo–6Nb–Zr; Ti–8Mo–5Nb–3Zr; Ti–8Mo–4Nb–2Zr; Ti–8Mo–4Nb–5Zr.	Arc melting	Ti-6Al-4V: 13–16%	Ti–8Mo–6Nb–4Zr: 15 %; Ti–8Mo–5Nb–3Zr: 21 %; Ti–8Mo–4Nb–2Zr: 15 %; Ti–8Mo–4Nb–5Zr: 26 %	Ti–8Mo–4Nb–5Zr > Ti–8Mo–5Nb–3Zr > Ti–8Mo–6Nb–4Zr > Ti–8Mo–4Nb–2Zr > Ti-6Al-4V.	NR	NR	NR
Niinomi et al.,1999[131]	Ti-13Nb-13zr; Ti-29Nb-13Ta-4.6Zr; Ti-16Nb-13Ta4-Mo; Ti-29Nb-13T; Ti-29Nb-13Ta-4Mo; Ti-29Nb-13Ta-2Sn; Ti-29Nb-13Ta-4.6Sn; Ti-29Nb-13Ta-6Sn.	Arc melting	Ti-6Al-4V ELI:: 15%	Ti-13Nb-13Zr: 20 %; Ti-29Nb-13Ta-4.6Zr: 40 %; Ti-16Nb-13Ta4-Mo: 65 %; Ti-29Nb-13T: 35 %; Ti-29Nb-13Ta-4Mo: 15 %; Ti-29Nb-13Ta-2Sn: 25 %; Ti-29Nb-13Ta-4.6Sn: 20 %; Ti-29Nb-13Ta-6Sn: 15 %.	Ti-16Nb-13Ta4-Mo > Ti-29Nb-13T > Ti-29Nb-13Ta-2Sn > Ti-13Nb-13Zr > Ti-29Nb-13Ta-4.6Sn > Ti-29Nb-13Ta-4.6Zr > Ti-29Nb-13Ta-6Sn > Ti-6Al-4V ELI.	NR	NR	NR
Li et al., 2023[208]	Ti-8Mo-3Sn; Ti-8Mo-5Sn; Ti-8Mo-7Sn; Ti-8Mo-9Sn; Ti-8Mo-11Sn; Ti-8Mo-13Sn; Ti-8Mo-15Sn	Arc melting	Ti-8Mo: 0.55 %	Ti-8Mo-3Sn: 0.48 %; Ti-8Mo-5Sn: 0.48 %; Ti-8Mo-7Sn: 0.44 %; Ti-8Mo-9Sn: 0.44 %; Ti-8Mo-11Sn: 0.33 %; Ti-8Mo-13Sn: 1.02 %; Ti-8Mo-15Sn: 1.56 %	Ti-8Mo-15Sn > Ti-8Mo-13Sn > Ti-8Mo-11Sn > Ti-8Mo-9Sn > Ti-8Mo-5Sn = Ti-8Mo-3Sn > Ti-8Mo.	NR	NR	NR
Kumar et al., 2009[209]	Ti–15Mo	NR	NR	NR	NR	CP–Ti: 175 HV; Ti–6Al–4V: 324 HV2)	Ti–15Mo: 238 HV	Ti–6Al–4V > Ti–15Mo > CP–Ti.

Kumar et al., 2019[210]	Ti-6Al-4V-2.5cu; Ti-29nb-13Ta-4.6Zr	NR	NR	NR	NR	Ti-6Al-4V: 298 ± 3.5 HV	Ti-6Al-4V-2.5Cu: 428 ± 4 HV; Ti-29Nb-13Ta-4.6Zr: 196 ± 2 HV	Ti-6Al-4V-2.5Cu > Ti-6Al-4V > Ti-29Nb-13Ta-4.6Zr.
Kopova et al., 2016[69]	Ti–35n–7Zr–5Ta–1Si; Ti–35n–7Zr–5Ta–2Fe; Ti–35n–7Zr–5Ta–0.5Si–1Fe; Ti–35n–7Zr–5Ta–0.5Si–2Fe; Ti–35n–7Zr–5Ta–1Si–1Fe	Arc melting	Ti–35N–7Zr–5Ta: 22%	Ti–35N–7Zr–5Ta–1Si: 12%; Ti–35N–7Zr–5Ta–2Fe: 27%; Ti–35N–7Zr–5Ta–0.5Si–1Fe: 14%; Ti–35N–7Zr–5Ta–0.5Si–2Fe: 15%; Ti–35N–7Zr–5Ta–1Si–1Fe: 8%	Ti–35N–7Zr–5Ta–2Fe > Ti–35N–7Zr–5Ta > Ti–35N–7Zr–5Ta–0.5Si–2Fe > Ti–35N–7Zr–5Ta–0.5Si–1Fe > Ti–35N–7Zr–5Ta–1Si–0.5Fe > Ti–35N–7Zr–5Ta–1Si–1Fe	NR	NR	NR
Cho et al., 2014[211]	Ti-14Mn	Arc Melting	Ti-10Mn: 12%	Ti-14Mn : 11%	Ti-10Mn > Ti-14Mn.	Ti-10Mn : 320 HV	Ti-14Mn :300 HV	Ti-10Mn > Ti-14Mn
Kuroda et al., 2020[68]	Ti-25Ta-10Zr; Ti-25Ta-20Zr; Ti-25Ta-30Zr; Ti-25Ta-40Zr	Arc melting	NR	NR	NR	Ti-25Ta:300 HV	Ti-25Ta-10Zr: 260 HV; Ti-25Ta-20Zr: 300 HV; Ti-25Ta-30Zr: 350 HV; Ti-25Ta-40Zr: 340 HV	Ti-25Ta-30Zr > Ti-25Ta-40Zr > Ti-25Ta > Ti-25Ta-20Zr > Ti-25Ta-10Zr.
Xie et al., 2015[90]	Ti–6Mo; Ti–8Mo; Ti–10Mo	Arc melting	37.4 %	Ti–6Mo: 30.8%; Ti–8Mo: 34.6%; Ti–10Mo: 32.3%.	NR	NR	NR	NR
Zhao et al., 2011[178]	Ti–30zr–2Mo; Ti–30Zr–5Mo; Ti–30Zr–6Mo; Ti–30Zr–7Mo; Ti–30Zr–8Mo	Argon casting with tri-arc furnace.	93%	Ti–30Zr–2Mo: 90%; Ti–30Zr–5Mo: 78%; Ti–30Zr–6Mo: 72%; Ti–30Zr–7Mo: 79%; Ti–30Zr–8Mo: 95%.	NR	NR	NR	NR
Wu et al., 2022[3]	Ti-24Nb-4Zr-1Mn; Ti-24Nb-4Zr-3Mn; Ti-24Nb-4Zr-5Mn	Arc melting	NR	NR	NR	Ti-24Nb-4Zr: 398 ± 6 HV	Ti-24Nb-4Zr-1Mn: 424 ± 11; Ti-24Nb-4Zr-3Mn: 437 ± 9; Ti-24Nb-4Zr-5Mn: 429 ± 5 HV	Ti-24Nb-4Zr-3Mn > Ti-24Nb-4Zr-5Mn > Ti-24Nb-4Zr-1Mn > Ti-24Nb-4Zr.
Arias-González et al., 2022[213]	Ti-42Nb	Laser-directed energy deposition	Ti-6Al-4V: 6-10%	Ti-42Nb : 11.6 % ± 4.0	Ti-42Nb > Ti-6Al-4V.	NR	NR	NR

Dai et al., 2013[220]	Ti−35Nb−4Sn−6Mo−3Zr; Ti−35Nb−4Sn−6Mo−6Zr; Ti−35Nb−4Sn−6Mo−9Zr; Ti−35Nb−4Sn−6Mo−12Zr; Ti−35Nb−4Sn−6Mo−15Zr	Arc fusion	Ti−35Nb−4Sn−6Mo: 15%	Ti−35Nb−4Sn−6Mo−3Zr: 13%; Ti−35Nb−4Sn−6Mo−6Zr: 12 %;Ti−35Nb−4Sn−6Mo−9Zr: 10 %; Ti−35Nb−4Sn−6Mo−12Zr: 11 %; Ti−35Nb−4Sn−6Mo−15Zr: 7%	NR	NR	NR	Ti−35Nb−4Sn−6Mo > Ti−35Nb−4Sn−6Mo−3Zr > Ti−35Nb−4Sn−6Mo−12Zr > Ti−35Nb−4Sn−6Mo−9Zr > Ti−35Nb−4Sn−6Mo−6Zr > Ti−35Nb−4Sn−6Mo−15Zr.
Afonso et al., 2017[2014]	Ti-63Fe-23Nb-8Sn6, Ti-60Fe-23Nb-8S-n9 Ti-66Fe-20Nb-8Sn6	Copper Mold Suction Casting, high-energy ball milling (HEBM), Laser Cladding	NR	NR	NR	NR	Ti-63Fe-23Nb-8Sn6: 488 ± 18, Ti-60Fe-23Nb-8Sn9: 608 ± 3,0 Ti-66Fe-20Nb-8Sn6: 417 ± 15	
Alberta et al., 2022[215]	(Ti–45Nb)96-4 Ga (Ti–45Nb)96–4Cu 45Nb)96-2 Ga–2Cu	Arc-melting	NR	NR	NR	Ti–45Nb: 147 (±3)	(Ti–45Nb)96-4 Ga; 231 (±10), (Ti–45Nb)96–4Cu: 202 (±5 45Nb)96-2 Ga–2Cu: 223 (±4)	(Ti–45Nb)96-4 Ga > (Ti–45Nb)96–4Cu > (45Nb)96-2 Ga–2Cu > Ti–45Nb.
Almeida et al., 2020[216]	Ti-12Mo-25Nb	Arc melting i	NR	NR	NR	CP Ti: 77 ± 0,2; Ti-6Al-4V: 336 ± 15	Ti-12Mo-25Nb : 207 ± 3	Ti-6Al-4V > Ti-12Mo-25Nb > CP Ti.
Awwaluddin et al., 2020[217]	Zr–6Mo–4Ti–2Y; Zr–6Mo–4Ti–3Y	Arc melting	NR	NR	NR	NR	Zr–6Mo–4Ti–1Y 461.8 Zr–6Mo–4Ti–2Y 335.1 Zr–6Mo–4Ti–3Y 301.5	NR
Bertrand et al., 2010[91]	Ti-25ta-25nb	Cold crucible semi-	NR	NR	NR	cpTi: 134	Ti-25ta-25nb: 145	Ti-25Ta-25Nb > cpTi.

		levitation melting						
Çaha et al., 2020[218]	Ti-15Nb Ti-40Nb.	NR	NR	NR	NR	Ti-6Al-4V: 350 ± 5 HV0.3	Ti-15Nb: 249 ± 3 HV0.3 Ti-40Nb: 262 ± 5 HV0.3	Ti-6Al-4V > Ti-40Nb > Ti-15Nb.
Correa et al., 2016[219]	Ti-15Zr-7.5Mo Ti-15Zr-15Mo.	Argon arc	NR	NR	NR	NR	Ti-15Zr-7.5Mo: 479-573 HV Ti-15Zr-15Mo: 398-573 HV	NR
Zhang et al., 2022[212]	Ti-15Mo	Cold crucible levitation melting	NR	NR	NR	Ti-6Al-4V: 334 Hv	Ti-15Mo(R): 334	Ti-6Al-4V =Ti-15Mo(R)
Sutowo et al.,2024[224]	Ti-6Mo-6Nb-4Sn, Ti-6Mo-6Nb-8Sn Ti-6Mo-6Nb-12Sn.	Arc melting	NR	NR	NR	NR	Ti-6Mo-6Nb: 419.72(as-cast), 353.88 VHN (H) Ti-6Mo-6Nb-4Sn: 327.0 VHN (as-cast), 295.5 VHN (H) Ti-6Mo-6Nb-8Sn: 277.5 VHN (as-cast), 350.5 VHN (H) Ti-6Mo-6Nb-12Sn: 330.7 VHN (as-cast), 397.4 VHN (H)	Ti-6Mo-6Nb-8Sn (As-Cast) > Ti-6Mo-6Nb-4Sn (H) > Ti-6Mo-6Nb (H) > Ti-6Mo-6Nb-8Sn (H) > Ti-6Mo-6Nb (As-Cast) > Ti-6Mo-6Nb-4Sn (As-Cast) > Ti-6Mo-6Nb-12Sn (As-Cast) > Ti-6Mo-6Nb-12Sn (H).
